# Two Birds with One Stone: Concurrent Ligand Removal and Carbon Encapsulation Decipher Thickness-Dependent Catalytic Activity

**DOI:** 10.1021/acs.nanolett.2c03181

**Published:** 2022-09-26

**Authors:** Kun Guo, Litao Chang, Ning Li, Lipiao Bao, Samir de Moraes Shubeita, Aliaksandr Baidak, Zhixin Yu, Xing Lu

**Affiliations:** † State Key Laboratory of Materials Processing and Die & Mould Technology, School of Materials Science and Engineering, Huazhong University of Science and Technology, Wuhan430074, People’s Republic of China; ‡ Department of Chemistry, The University of Manchester, ManchesterM13 9PL, United Kingdom; § Shanghai Institute of Applied Physics, Chinese Academy of Sciences, Shanghai201800, People’s Republic of China; ∥ Dalton Cumbrian Facility, The University of Manchester, CumbriaCA24 3HA, United Kingdom; ⊥ Institute of New Energy, School of Chemistry and Chemical Engineering, Shaoxing University, Shaoxing312000, People’s Republic of China; # Department of Energy and Petroleum Engineering, University of Stavanger, 4036Stavanger, Norway

**Keywords:** encapsulation, shell thicknesses, molybdenum disulfide, electrocatalysts, hydrogen evolution reaction

## Abstract

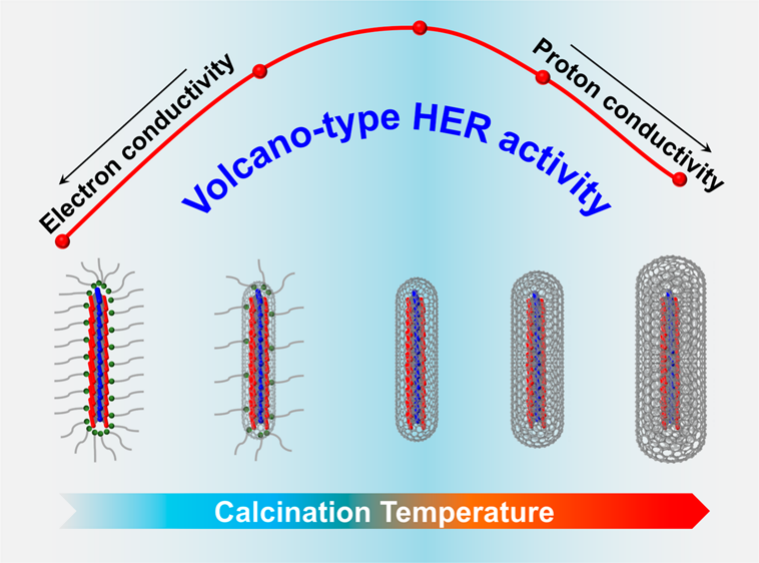

A carbon shell encapsulating a transition metal-based core has emerged as an intriguing type of catalyst structure, but the effect of the shell thickness on the catalytic properties of the buried components is not well known. Here, we present a proof-of-concept study to reveal the thickness effect by carbonizing the isotropic and homogeneous oleylamine (OAm) ligands that cover colloidal MoS_2_. A thermal treatment turns OAm into a uniform carbon shell, while the size of MoS_2_ monolayers remains identical. When evaluated toward an acidic hydrogen evolution reaction, the calcined MoS_2_ catalysts deliver a volcano-type activity trend that depends on the calcination temperature. Rutherford backscattering spectrometry and depth-profiling X-ray photoelectron spectroscopy consistently provide an accurate quantification of the carbon shell thickness. The same variation pattern of catalytic activity and carbon shell thickness, aided by kinetic studies, is then persuasively justified by the respective limitations of electron and proton conductivities on the two branches of the volcano curve.

A pressing issue recognized by the catalysis community is that the applied solid catalysts often experience uncontrollable structural and compositional variations under harsh reaction conditions.^1−6^ One solution that is being strongly pursued is to cover the catalysts with a carbon shell that protects the components beneath. These peculiar types of core–shell catalysts have demonstrated fascinating advantages, such as resistances to agglomeration, corrosion, and oxidation, reaction selectivity control, and an electron transfer effect.^7−9^ However, the influence of the carbon shell structure on the buried core has been poorly elaborated due inevitably to the challenge of fine-tuning the shell structural factors while retaining identical inner cores.

One of the intriguing structural factors of a carbon shell is its thickness along the radial direction. A critical, yet often ignored, question is how the thickness of the carbon shell dictates the catalytic behavior, especially from the perspective of proton-coupled electron transfer that lays the foundation of many electrochemical reactions.^10,11^ In the cases of single- and few-layer graphene, previous studies have verified that protons can penetrate through, allowing their accessibility to the core materials beneath.^12−16^ However, a carbon shell that is too thick may block the reactants from accessing the active sites and detain the products from diffusing away. The shell thickness also affects the synergistic interactions between the metal core and the outer few carbon layers that are catalytically relevant. Theoretical calculations by Bao and co-workers revealed that the electron transfer between the carbon shell and metal core gradually intensifies and remains steady when the shell is thicker than three graphene layers.^17^ Therefore, the effect of shell thickness deserves considerable attention to precisely evaluate the catalysts of interest, but relevant in-depth studies on this aspect are still lacking.

The key to deciphering the shell thickness effect is the controllable synthesis of carbon-encapsulated materials with well-defined structural parameters. Furnace pyrolysis is the prevailing route to yield metal-based cores embedded in carbon shells.^18−20^ Nonetheless, it is extremely difficult to control the structures and compositions of both the core and shell because the process is largely empirical, making the precise discrimination of the catalytic contributions of core and shell difficult.^21−23^ Colloidal synthesis in the presence of organic ligands has demonstrated its competence to tailor-make low-dimensional transition metal-based nanomaterials.^24−26^ Calcination is often required to recover ligand-stripped catalysts, but what indeed occurs is that calcination does not remove all the ligands but turns part of them into carbon layers covering inner cores.^27−29^ In light of the colloidal nanomaterials with narrow size distributions and homogeneous ligand coverage, calcination of ligand-capped nanomaterials holds the potential to solve two problems at one time. On one hand, the detrimental ligands that sterically and chemically block the metal active sites can be efficiently eliminated. On the other hand, the isotropic and homogeneous surface ligands can be converted into uniform carbon shells with a well-defined and adjustable thickness.^30^ The resulting products thus serve as a desirable prototype to investigate the thickness effect of the carbon shell on the catalytic properties of encased cores.^31^

Drawing on the preceding synthesis of ligated MoS_2_ monolayers,^32^ we take one step further by calcining the monolayers at controlled temperatures in the range of 200–600 °C to carbonize the ligands without affecting the size of the MoS_2_ core. The hydrogen evolution reaction (HER) activity in acids of the calcined products is found to be dependent on the calcination temperature, forming a volcano-type activity curve. Microscopic and spectroscopic characterizations unambiguously disclose the formation of a carbon shell around the MoS_2_ monolayers. The relative thickness of the carbon shell is further quantified by Rutherford backscattering spectrometry and depth-profiling X-ray photoelectron spectroscopy. The results not only are closely in accord with each other but also decipher the volcano-type thickness-dependent activity, which is attributed to the tradeoff between electron conductivity (ligand coverage) and proton conductivity (mass transfer).

Figure 1 illustrates the colloidal synthesis and postcalcination treatments of unsupported MoS_2_ and Super P carbon supported MoS_2_ (MoS_2_/C). Oleylamine (OAm) functions as both the solvent and the surfactant.^33−36^ The applicability of ethanol/hexane washing for OAm removal is first tested. OAm-ligated Pd and Ni_2_P nanoparticles are also prepared following similar procedures (Figure S1). Attenuated total reflection Fourier transform infrared (ATR-FTIR) spectra of the obtained materials and reference chemicals confirm the existence of OAm according to the absorption peaks arising from various vibrational modes in the OAm molecule (Figure S2). After solvent washing, OAm is significantly eliminated for Pd and Ni_2_P but barely removed for MoS_2_-AP, indicating the strong coordination between OAm and MoS_2_.

**Figure 1:**
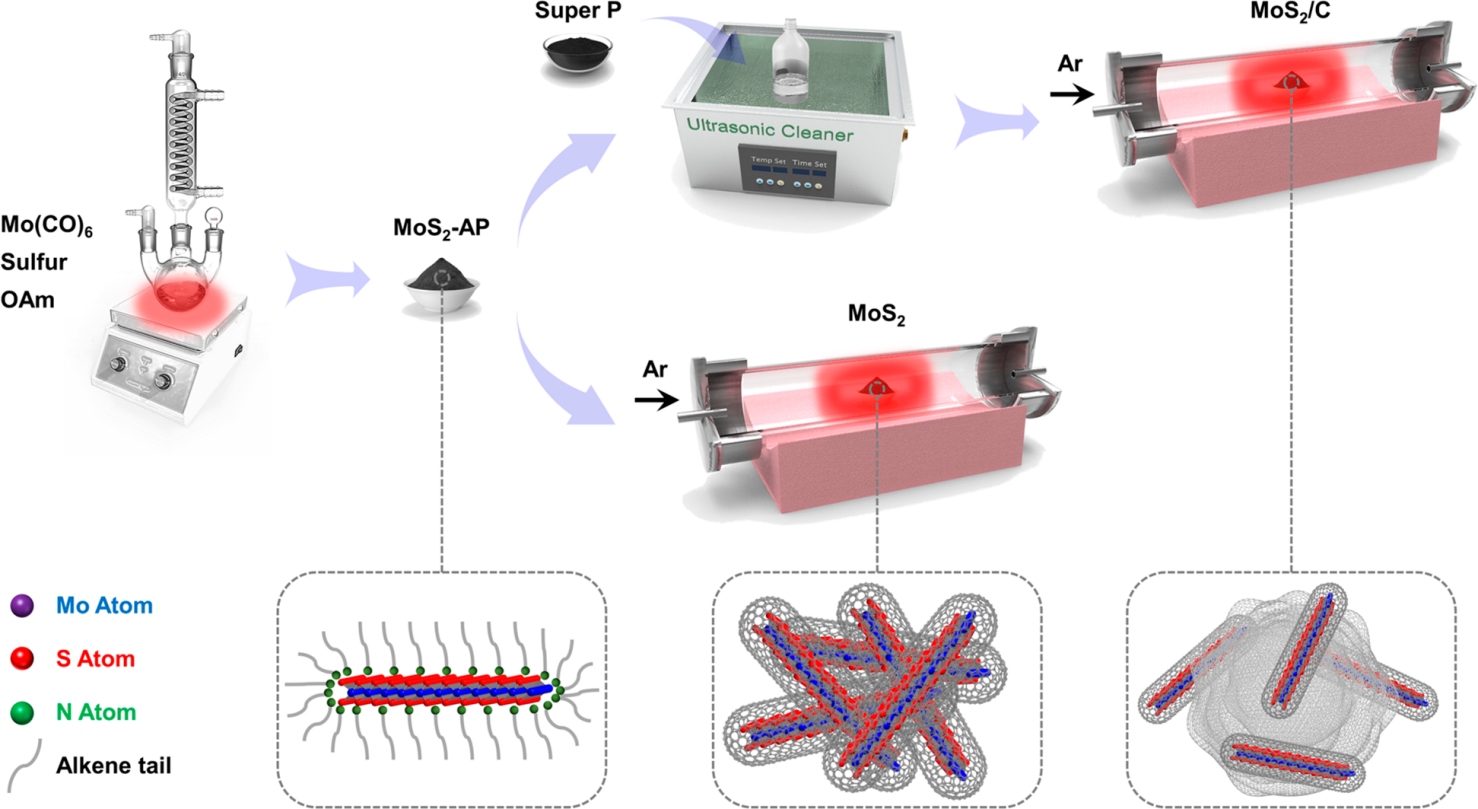
Schematic illustration of the colloidal synthesis of OAm-ligated MoS_2_ monolayers and the postcalcination to produce MoS_2_/C and MoS_2_.

A transmission electron microscope (TEM) image of MoS_2_-AP is shown in Figure 2a. Randomly oriented dark lines are discerned, which stem from the side view of disordered MoS_2_ monolayers. A statistical count and a corresponding Gaussian fit of at least 200 monolayers give an ultrafine lateral size of 4.3 ± 1.1 nm. The X-ray diffraction (XRD) patterns of MoS_2_-AP and MoS_2_/C-AP (Figure S3) confirm the hexagonal 2H-MoS_2_ phase (JCPDS card no. 37-1492). The broad characteristic peaks at 32.7 and 58.3°, corresponding to the (100) and (110) in-plane diffraction, respectively, manifest the low crystallinity of MoS_2_. The absence of the (002) characteristic peak at 14.3° in Figure S4 further demonstrates the monolayer structure, in stark contrast to the highly crystalline commercial MoS_2_ (c-MoS_2_). The (004) peak originally located at 29.0° of standard 2H-MoS_2_ is shifted to 18.5°, indicating an extended interplanar spacing along the *c* axis according to Bragg’s law.

**Figure 2:**
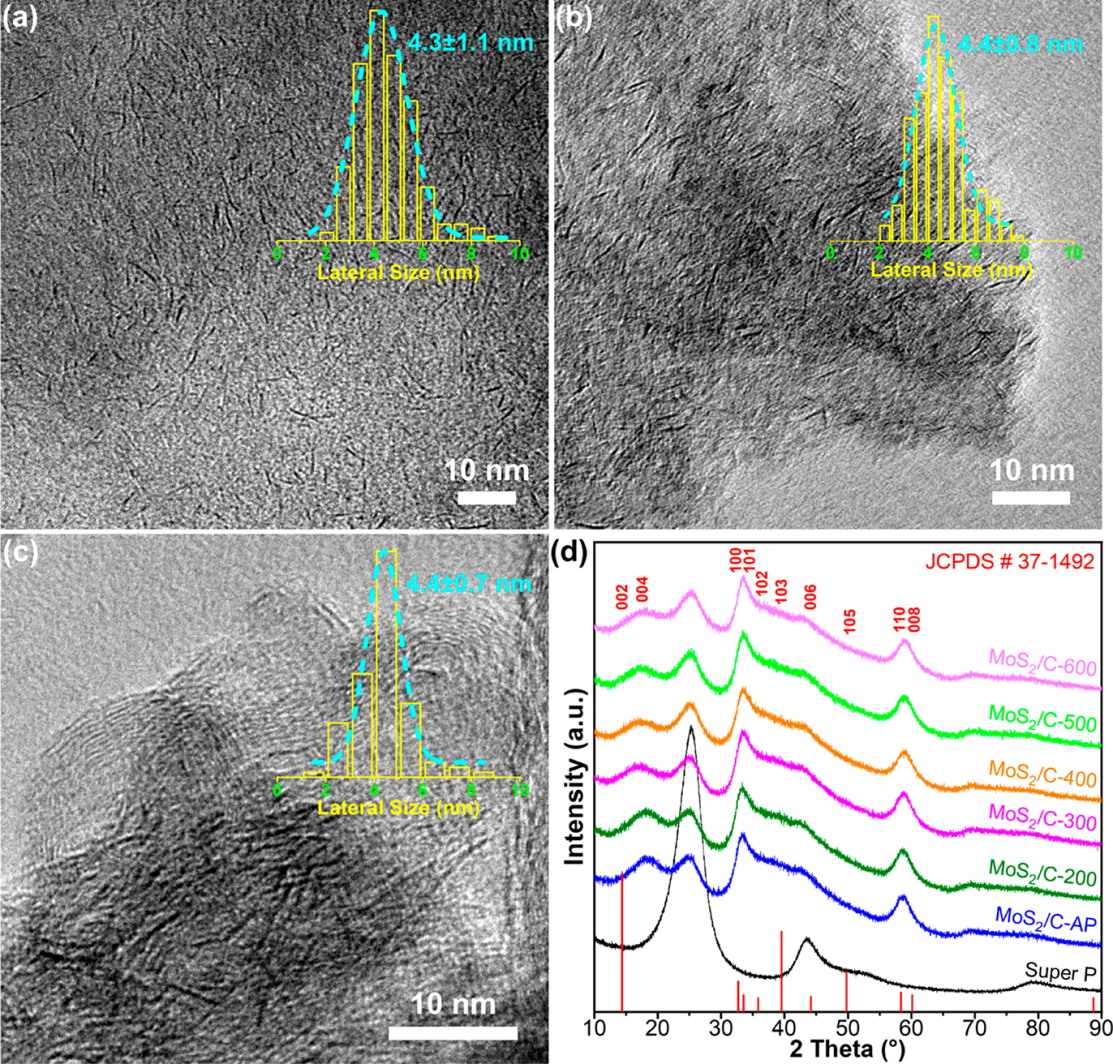
TEM images and the corresponding lateral size distributions of MoS_2_-AP (a), MoS_2_-400 (b), and MoS_2_/C-400 (c). (d) XRD patterns of the calcined MoS_2_/C, MoS_2_/C-AP, and Super P.

To remove the ligands, we calcined the MoS_2_-AP and MoS_2_/C-AP at 200–600 °C. Analogous dark lines in MoS_2_-400 (Figure 2b) are observed with an almost identical lateral size. Moreover, the monolayers are attached to an amorphous carbon substrate resulting from OAm carbonization (*vide infra*). In comparison, MoS_2_/C is also annealed at 400 °C (Figure 2c). The spherical carbon particles of Super P with clear lattice fringes are discerned alongside the dark lines of MoS_2_. Again, the average lateral size of MoS_2_ monolayers barely vary from those of MoS_2_-AP and MoS_2_-400. XRD patterns in Figure 2d indeed show that the structure and size of MoS_2_ are well retained at calcination temperatures of even up to 600 °C. Super P itself is very stable during sintering (Figure S5). This result is hence linked to the carbon shell that affords high sintering resistance and structural stability. Scanning electron microscopy unveils the consistent surface morphology of Super P and the calcined MoS_2_/C (Figure S6), distinct from that of the calcined MoS_2_ (Figure S7).

Figure 3a shows the ATR-FTIR spectra of calcined and as-prepared MoS_2_/C. The prominent absorption peaks from the most representative aliphatic tail of OAm, enlarged in the right panel, hardly appear at 400 °C and above, manifesting the complete transformation of OAm. To preclude the effect of Super P carbon, we also compare the ATR-FTIR spectra of MoS_2_-AP and calcined MoS_2_ (Figure 3b). It is again found that a threshold of 400 °C is necessary for the total elimination of OAm ligands. The Raman spectra of supported MoS_2_ in Figure 3c present two major peaks at 1350 and 1599 cm^–1^, ascribed to the D and G bands of graphitic structures, respectively. The Raman spectra of the unsupported MoS_2_ are displayed in Figure 3d. For both MoS_2_-200 and MoS_2_-AP, D and G bands are not observed. When the temperature is elevated to 300 °C, both D and G bands appear, directly evidencing the carbon formation.^37^ The higher graphitization degree of the resultant carbon calcined at a higher temperature is reflected in the increasing *I*_D_/*I*_G_ ratios. Moreover, two minor peaks located at lower Raman shifts, corresponding to the  and  vibrational modes of lamellar MoS_2_ (Figure S8), are discerned. Compared to c-MoS_2_, both  and  bands are shifted for all the supported and unsupported samples, especially those annealed at relatively high temperatures. The red shift of the  band is anticipated due to the monolayer structure.^38,39^ However, the  band is not blue-shifted, which is interpreted as being a disturbance of the defective layer structure and ultrafine lateral size of MoS_2_.^40−42^

**Figure 3:**
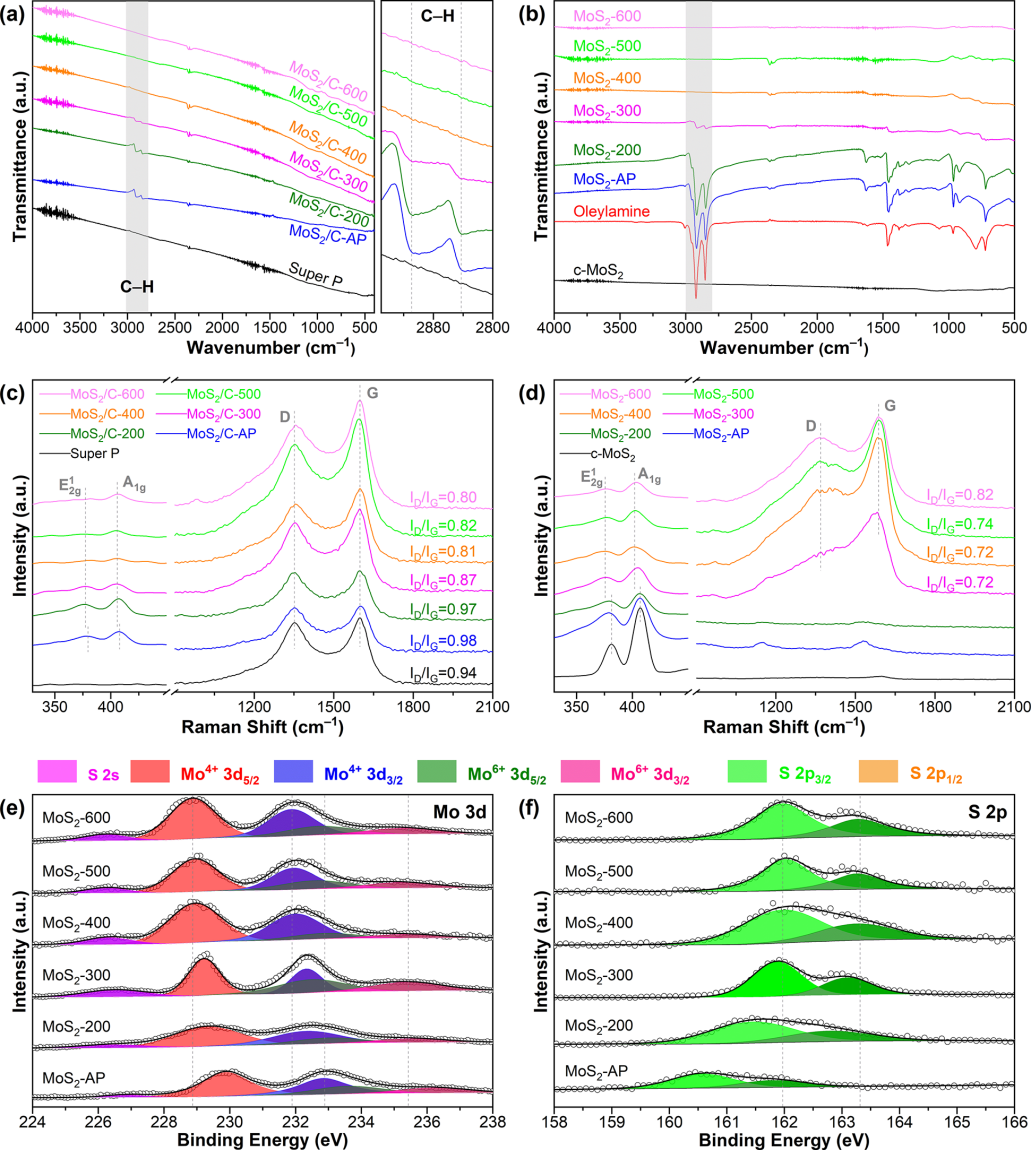
ATR-FTIR and Raman spectra of (a, c) the calcined, as-prepared MoS_2_/C and Super P, and (b, d) the calcined, as-prepared MoS_2_ and c-MoS_2_. The right panel of (a) is an enlargement of the highlighted region that represents the C–H bond stretching vibration. The corresponding *I*_D_/*I*_G_ ratios are indicated in (c, d). High-resolution XPS spectra of Mo 3d (e) and S 2p (f) regions of the calcined and the as-prepared MoS_2_.

A depth-profiling X-ray photoelectron spectroscopy (XPS) analysis coupled with 300 eV Ar^+^ etching is further performed to probe the elemental composition and speciation at different depths. Commercial c-MoS_2_ is included as a reference (Figure S9). All of the full survey XPS spectra of the calcined MoS_2_ at three etching stages identify the elements C, N, O, S, and Mo (Figure S10). The inconspicuous C 1s peak changes of each sample before and after etching can be ascribed to the random orientation of MoS_2_ monolayers throughout the carbon shell (Figure S11). In contrast, Figure S12 shows that the C 1s peak is the smallest for MoS_2_-400 among the calcined MoS_2_ etched for 90 s. The N 1s region is close to that of Mo 3p, and the XPS spectra are then deconvoluted into Mo^4+^ 3p_3/2_, Mo^6+^ 3p_3/2_, and N 1s (Figure S13). The N 1s peak shrinks with an increase in the calcination temperature and disappears at 400 °C, verifying the complete removal of OAm. The Mo 3d and S 2p XPS spectra after 90 s of etching are shown in Figure 3e,f, respectively. As the calcination temperature increases, all of the doublet peaks of Mo 3d move to lower binding energies, indicating that fewer 3d electrons are transferred from MoS_2_ to OAm as OAm is being gradually removed. After OAm removal, the Mo^4+^ 3d_5/2_ and 3d_3/2_ peaks are positioned at almost the same binding energy as that of c-MoS_2_ (Figure S14). In Figure 3f, the S 2p_3/2_ and 2p_1/2_ doublets are shifted to higher binding energy, implying that fewer electrons are withdrawn from S atoms as OAm ligands are eliminated. This is in contradiction with the fact that more 3d electrons are available by Mo as fewer are transferred to N. It is inferred that N–H···S hydrogen bonds could be formed to allow electron withdrawal from adjacent H atoms to low-valent S atoms. Removal of OAm thus results in the upward shifting of the binding energy of S 2p electrons to the same positions as in c-MoS_2_ (Figure S14).

The HER activity of the calcined MoS_2_/C is assessed in Ar-saturated 0.5 M H_2_SO_4_. Figure 4a shows the *iR*-compensated linear sweep voltammetry (LSV) polarization curves of the catalysts being studied. The overpotential at a current density of 10 mA cm^–2^ (η_10_) is compared in the upward axis of the radar chart in Figure 4b. Despite the ultrafine lateral size of MoS_2_/C-AP, its η_10_ value is found to be close to that of c-MoS_2_/C, indicating the poor HER activity of MoS_2_/C-AP. Calcination of MoS_2_/C-AP at 200 and 300 °C leads to a gradually reduced η_10_ value and improved activity. When the calcination reaches 400 °C, η_10_ is reduced by 280 and 130 mV compared to the values for Super P and MoS_2_/C-AP, respectively. However, at even higher temperatures, η_10_ starts to increase for MoS_2_/C-500 and MoS_2_/C-600, indicating their deteriorated HER activities. Figure 4c shows the Tafel plots, and the derived Tafel slopes are presented in the rightward axis of Figure 4b. MoS_2_/C-400 delivers the smallest Tafel slope of 76 mV dec^–1^. The other catalysts annealed at lower or higher temperatures have values larger than 118 mV dec^–1^. As elaborated in Note S1, the rate-determining step (RDS) for c-MoS_2_/C should be the Volmer step that involves electron transfer and proton adsorption at the active sites (H*), in agreement with the facts that hydrogen adsorption to the 2H-MoS_2_ basal plane is thermodynamically unfavorable and electron transfer to semiconductive 2H-MoS_2_ is slow.^43,44^ For the catalysts calcined at temperatures either lower or higher than 400 °C, the Volmer step is also the RDS. In contrast, the Heyrovsky step is the RDS for MoS_2_/C-400, manifesting the sluggish electrochemical desorption of H* on the active sites.^45−48^ It is thus deduced that the electronic structure of MoS_2_ has been modulated by carbon encapsulation to favor the adsorption of H*.

**Figure 4:**
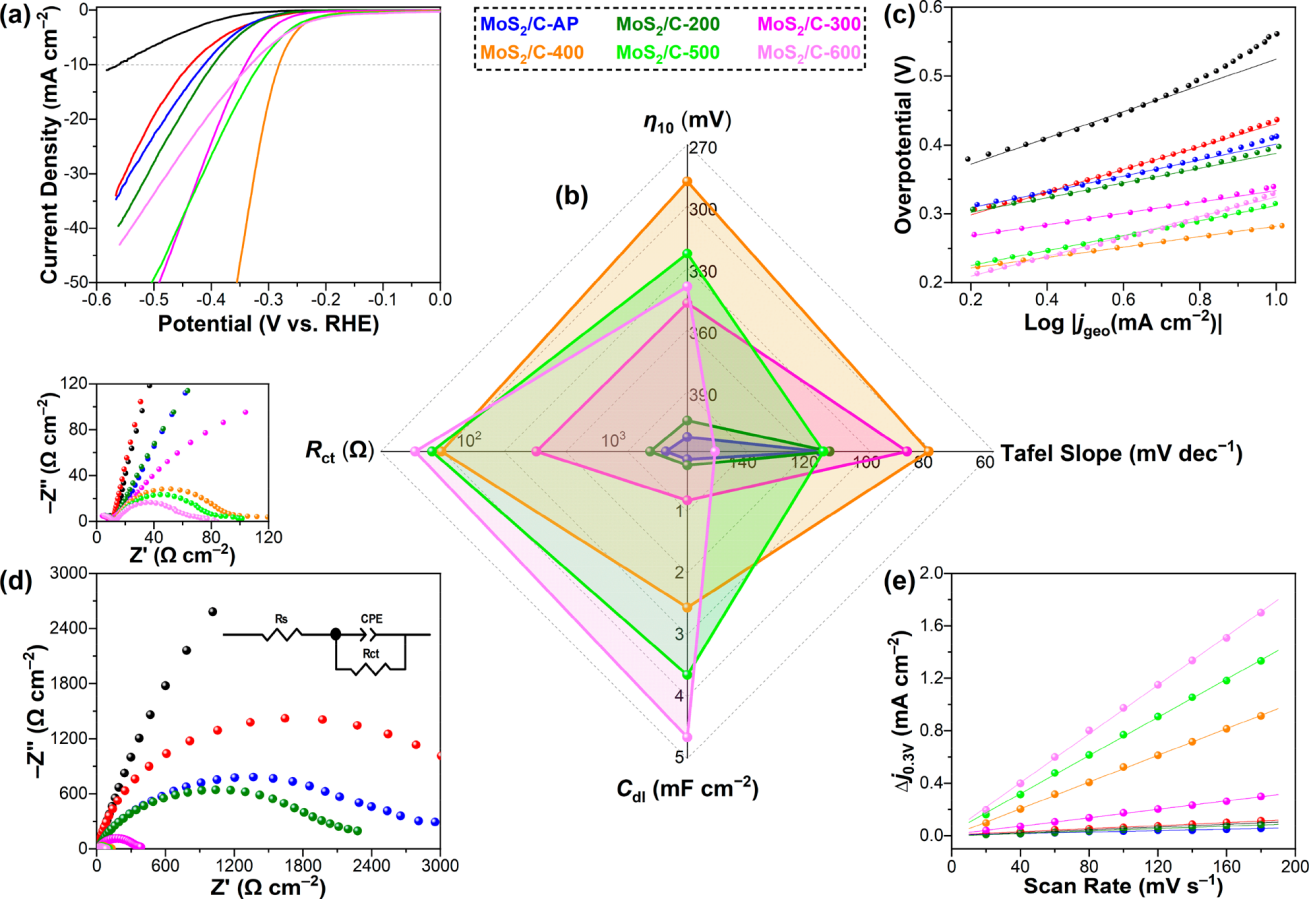
(a) *iR*-compensated LSV polarization curves, (b) a radar chart using η_10_, Tafel slope, *C*_dl_, and *R*_ct_ as four axes, (c) Tafel plots, (d) Nyquist plots, and (e) differences in current density ((*j*_anodic_ – *j*_cathodic_)/2) at 0.3 V_RHE_ plotted against the scan rate of calcined MoS_2_/C, MoS_2_/C-AP, c-MoS_2_/C, and Super P. The HER is tested in Ar-saturated 0.5 M H_2_SO_4_. The inset in (d) is the equivalent circuit model for fitting the impedance spectra. The graph above (d) is an enlargement of the Nyquist plots. All parts of the figure share the same legend based on the line/dot coloring.

Electrochemical impedance spectroscopy (EIS) measurements are carried out for all the catalysts, and the Nyquist plots are presented in Figure 4d. The solution resistance *R*_s_ is determined to be around 10 Ω, which is used for *iR* compensation. The charge transfer resistance *R*_ct_ represented by the semicircular diameter is compared in the leftward axis of Figure 4b. With an increase in calcination temperature, *R*_ct_ of the calcined MoS_2_/C gradually decreases, indicating a reduced resistance in electron transfer at the electrode/electrolyte interfaces. This agrees with the higher graphitization degree or electrical conductivity of the carbon shell obtained at higher temperatures, as revealed by the Raman analysis in Figure 3d. The double-layer capacitance (*C*_dl_) is calculated to assess the electrochemically active surface area. According to the non-Faradaic CV curves (Figure S15), the capacitive current density at 0.3 V_RHE_ is plotted against the scan rate (Figure 4e). After linear fitting, the obtained *C*_dl_ is compared in the downward axis of Figure 4b. It is found that the *C*_dl_ value of calcined MoS_2_/C increases constantly with the calcination temperature. Given that carbon materials with a high specific surface area also exhibit capacitive behavior, the larger *C*_dl_ value of MoS_2_/C sintered at higher temperatures should be largely due to the carbon shell with a greater thickness. Nonetheless, according to the colored areas linking four key performance indicators in the radar chart of Figure 4b, a better catalyst should cover a larger area and MoS_2_/C-400 thus has the best performance among all of the catalysts.

The preceding structural characterizations and catalytic performance hint that the shell thickness of MoS_2_/C varies with the calcination temperature. The Rutherford backscattering spectrometry (RBS, Note S2) is then employed for quantification of the carbon shell thickness of calcined unsupported MoS_2_, in addition to the depth-profiling XPS. Graphite bulk and thin gold films are utilized to calibrate the energy offset and energy per channel (Figure S16). Figure 5a shows the experimental and simulated RBS spectra using the SIMNRA code.^49^ All of the materials present stair-shaped spectra featured by typical bulk samples, indicating their relatively high thicknesses deposited on silicon wafers. The simulated spectra of single-phase MoS_2_ and carbon films with different thicknesses and layer configurations are also given in Figure S17. The front edges at channels of 457, 657, 981, 1112, and 1564, representing the elements C, O, Si, S, and Mo, respectively, are indicated. The sharp peak at channel 451 is ascribed to non-Rutherford elastic backscattering. N is not detected due, probably to its low intensity and signal overlapping. Provided that the surface of calcined MoS_2_ should be carbon layers, shifting of the front edges of buried Mo and S elements should be expected due to energy loss of incident ion particles traveling through the carbon layers. However, such shifting is not obvious in Figure 5a, indicating that the carbon layer has a thickness of several nanometers, as revealed by the simulations in Figure S17. The RBS spectra of blank silicon wafer show no major C peak, ruling out the potential origin of adventitious carbon from the solvent or other organic impurities (Figure S18).

**Figure 5:**
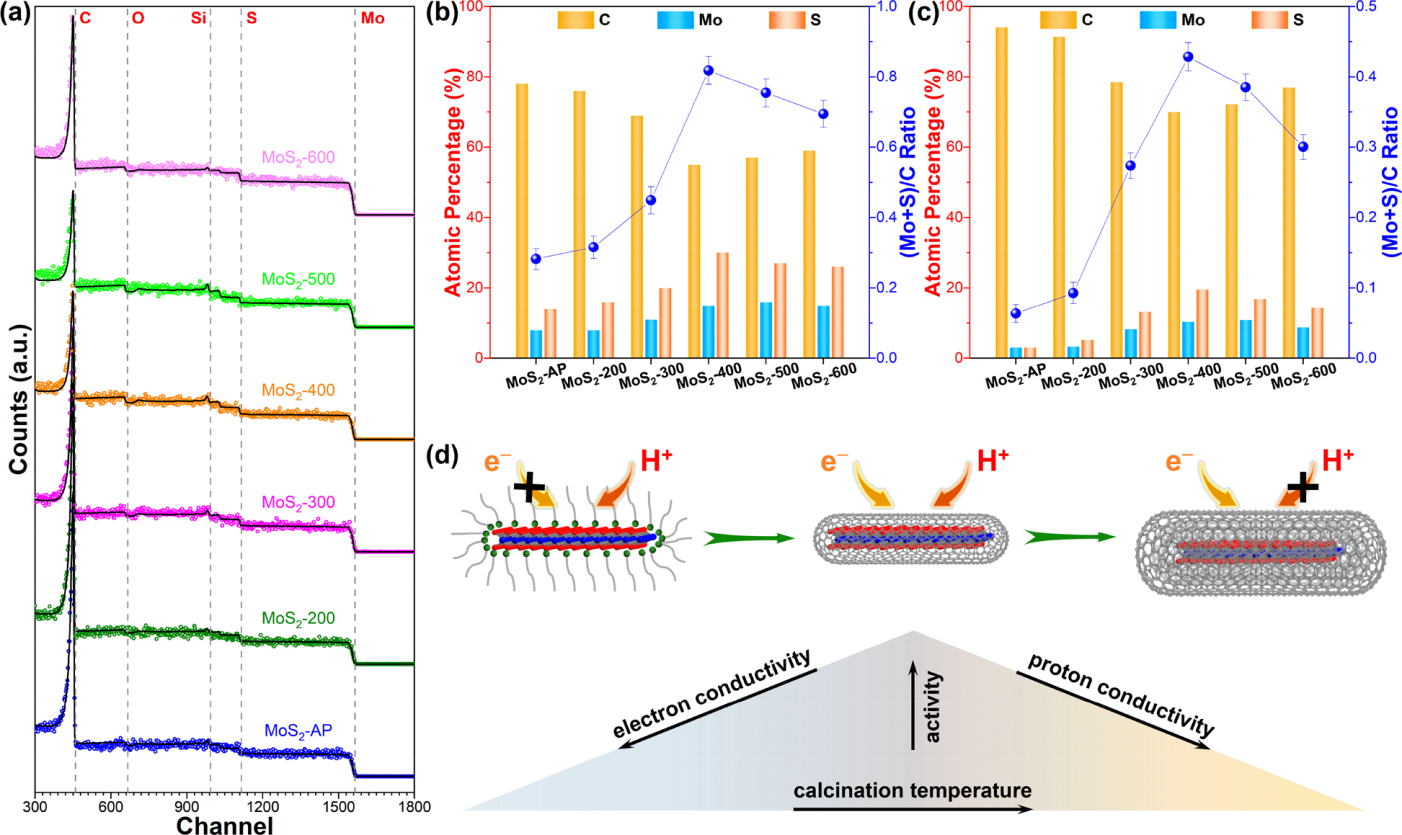
(a) Experimental and simulated RBS spectra and normalized atomic percentages of C, Mo, S, and measured (Mo + S)/C atomic ratios derived from RBS (b) and XPS (c) of the calcined and as-prepared MoS_2_. The colored circles and solid lines in (a) refer to the experimental and simulated data, respectively. Channels corresponding to the front edges of C, O, Si, S, and Mo elements are indicated by dashed lines. (d) Schematic illustration of the effect of surface encapsulation on the electron and proton conductivities of identically sized MoS_2_ monolayers.

Based on the fitted data, the average atomic percentage normalized to C, Mo, and S atoms together with the (Mo + S)/C atomic ratio (Table S1), which quantifies the relative thickness of the carbon shell, is plotted in Figure 5b. MoS_2_-AP possesses a high C content that originates from the OAm ligands. Upon calcination, OAm ligands are vaporized, accounting for the ever-decreasing C contents of MoS_2_-200, MoS_2_-300, and MoS_2_-400. At temperatures of 500 and 600 °C, carbonization of OAm ligands becomes the dominant process, leading to higher C contents in comparison to MoS_2_-400. The smallest carbon shell thickness is then achieved at 400 °C. Furthermore, the relative thickness is also derived from the XPS data (Table S2). As presented in Figure 5c, the relative thickness derived from XPS follows the exact same variation as the RBS results. In addition to this observation, the Mo/S ratios are in good accord with the stoichiometric ratio of MoS_2_.

Given the above, a rational explanation for the volcano-type HER activity trend can be made, which is briefly illustrated in Figure 5d. First, TEM and XRD results show that the lateral size and monolayer structure of calcined MoS_2_ are well preserved, excluding the MoS_2_ size effect on HER activity. As collectively revealed by multiple techniques, MoS_2_/C-AP and MoS_2_/C-200 are strongly capped by OAm, deactivating the surface-active sites for the HER. A Tafel analysis indicates that the Volmer step that involves proton-coupled electron transfer is rate-determining for these catalysts. Considering the large *R*_ct_ from EIS measurements, we believe that the low electron conductivity should be held accountable for the poor activity of these OAm-ligated catalysts. Removal and translation of OAm by calcination gradually release the active sites and thus result in the enhanced HER activity of MoS_2_/C-300 and MoS_2_/C-400. In addition, the carbon shell tunes the electronic structure of the catalyst that eventually favors the adsorption of H*, changing the RDS to the Heyrovsky step. Further elevation of the calcination temperature leads to an intensified carbonization of OAm, and MoS_2_ monolayers are thus coated by thicker shells. The RDS for both MoS_2_/C-500 and MoS_2_/C-600 is then shifted back to the Volmer step. By virtue of the improved graphitization degree and reduced *R*_ct_ from EIS results, their degenerative kinetics should be attributed to the inadequate proton conductivity rather than the electron conductivity as above.

To summarize, we have investigated the effects of thermal calcination on the ligand carbonization and carbon shell thickness on the catalytic properties of MoS_2_ by utilizing OAm-ligated MoS_2_ monolayers. OAm is found to be gradually pyrolyzed into a carbon shell as the calcination temperature increases. When tested toward an acidic HER, the calcined catalysts deliver a volcano-type activity trend with MoS_2_/C-400 presenting the highest activity. RBS and depth-profiling XPS results indicate that the best-performing MoS_2_/C-400 is neither capped by excessive OAm ligands nor covered by thick carbon layers, in sharp contrast to other catalysts calcined at higher or lower temperatures. In this case, the proton and electron transfer are both improved to surmount the slow kinetics of the Volmer step. This study not only gives attention to the judicious selection of calcination conditions for ligand removal but also unambiguously uncovers the shell thickness effect of core–shell structured catalysts on the catalytic activity.

## Supporting Information Available

Experimental section, briefs of Tafel analysis and RBS, normalized elemental contents, and additional XRD, ATR-FTIR, SEM, XPS, CV, and RBS data (PDF)
