# Supplementary material for: Two Birds with One Stone: Concurrent Ligand Removal and Carbon Encapsulation Decipher Thickness-Dependent Catalytic Activity
Source: Nano Lett. 2022 Sep 26;22(21):8763–70. doi: 10.1021/acs.nanolett.2c03181 (PMC9650766; doi:10.1021/acs.nanolett.2c03181)
Supplement: Supplementary file 1 — nl2c03181_si_001.pdf [file nl2c03181_si_001.pdf]

Two Birds with One Stone: Concurrent Ligand Removal and Carbon Encapsulation Decipher Thickness-Dependent Catalytic Activity

Kun Guo,[†,‡] Litao Chang,[‖] Ning Li,[†] Lipiao Bao,[†] Samir de Moraes Shubeita,[+] Aliaksandr Baidak,[‡,+] Zhixin Yu,[*,#] and Xing Lu[*,†]

[†]State Key Laboratory of Materials Processing and Die & Mould Technology, School of Materials Science and Engineering, Huazhong University of Science and Technology, Wuhan 430074, People's Republic of China

[‡]Department of Chemistry, The University of Manchester, Manchester M13 9PL, United Kingdom

[§]Shanghai Institute of Applied Physics, Chinese Academy of Sciences, Shanghai 201800, People's Republic of China

[‖]Dalton Cumbrian Facility, The University of Manchester, Cumbria CA24 3HA, United Kingdom

[⊥]Institute of New Energy, School of Chemistry and Chemical Engineering, Shaoxing University, Shaoxing 312000, People's Republic of China

[#]Department of Energy and Petroleum Engineering, University of Stavanger, 4036 Stavanger, Norway

Corresponding Authors:

Z. Yu: zhixin.yu@uis.no

X. Lu: lux@hust.edu.cn

Table of Contents

Experimental Section 3
Note S1. Tafel analysis of HER kinetics 7
Note S2. Rutherford backscattering spectrometry (RBS) 10
Table S1. Normalized elemental contents in atomic percentage of the calcined MoS_2 and MoS_2-AP derived from the RBS analysis. 12
Table S2. Normalized elemental contents in atomic percentage of the calcined MoS_2 and MoS_2-AP derived from the depth-profiling XPS after 90s etching. 13
Figure S1. XRD patterns of the as-prepared Pd (a) and Ni_2P (b) NPs indexed to JCPDS card No. 87-0643 and 65-1989, respectively 14
Figure S2. ATR-FTIR spectra of the as-prepared Pd and Ni_2P NPs, MoS_2-AP, and reference chemicals of $Pd(acac)_2$, $Ni(acac)_2$, TOP, and OAm. 15
Figure S3. XRD patterns of Super P, MoS_2/C-AP, and MoS_2-AP indexed to the hexagonal 2H-MoS_2 with the JCPDS card No. 37-1492. 16
Figure S4. XRD pattern of the c-MoS_2 indexed to the hexagonal 2H-MoS_2 with the JCPDS card No. 37-1492. 17
Figure S5. XRD patterns (a) and Raman spectra (b) of pristine and calcined Super P carbon black at 600 °C for 4 h in Ar. 18
Figure S6. SEM images of Super P (a), MoS_2/C-AP (b), MoS_2/C-400 (c), and MoS_2/C-600 (d). Scale bar: 100 nm. 19
Figure S7. SEM images of MoS_2-AP (a), MoS_2-400 (b), and MoS_2-600 (c). Scale bar: 1 µm. 20
Figure S8. Schematic illustration of the E_{2g}^1 and A_{1g} vibrational modes of MoS_2. 21
Figure S9. Depth-profiling XPS spectra of the Mo 3p region of c-MoS_2 etched by 300 eV Ar^+: unetched (top), after 45 s etching (middle), and after 90 s etching (bottom). The same intensity scale is set for all the samples. 22
Figure S10. Depth profiling XPS full survey of the calcined MoS_2 etched by 300 eV Ar^+. The red, blue, and green lines represent unetched, after 45s etching, and after 90s etching, respectively. 23
Figure S11. Schematic illustration of the encapsulated MoS_2 monolayers randomly oriented and distributed within the C shell. The depth of 3λ (λ is the electron mean free path) that most photoelectrons can escape and be detected by XPS is marked. 24
Figure S12. High-resolution XPS spectra of C 1s region of the calcined MoS_2 etched by 300 eV Ar^+ for 90 s for removal of adventitious carbon. The same intensity scale is set for all of the samples. 25
Figure S13. Depth profiling XPS spectra of the Mo 3p/N 1s region of the calcined MoS_2 etched by 300 eV Ar^+. In each panel, the top, middle, and bottom spectra represent unetched, after 45 s etching, and after 90 s etching, respectively. 26
Figure S14. High-resolution XPS spectra of Mo 3d and S 2p regions of c-MoS_2 after 90s etching by Ar^+. Mo 3d peak is resolved into spin-orbit doublets, Mo^{4+} $3d_{5/2}$ and $3d_{3/2}$, at 229.0 and 232.2 eV. S 2p peak is resolved into spin-orbit doublets, S $2p_{3/2}$ and $2p_{1/2}$, at 161.9 and 163.1 eV 27
Figure S15. Cyclic voltammograms of Super P (a), c-MoS_2/C (b), MoS_2/C-AP (c) and MoS_2/C calcined at temperatures of 200 (d), 300 (e), 400 (f), 500 (g), and 600 (h) °C in the potential range of 0.25–0.35 V_{RHE} and the scan rate range of 20–180 mV s^{-1}. 28
Figure S16. Experimental and simulated RBS spectra of thick graphite and thin gold film references. The areal thickness is indicated on the right. The substrate of gold film is not simulated. 29
Figure S17. Simulated RBS spectra of MoS_2 films with varied thicknesses of 200, 2000, and 40000 TFU, carbon film with a thickness of 100 TFU, and carbon films with thicknesses of 100, 1000 TFU on top of a 40000 TFU MoS_2 film. 30
Figure S18. Experimental and simulated RBS spectra of blank silicon wafer deposited by pure solvent. The areal thickness is indicated on the right. Only tiny amount of adventitious carbon is detected. 31

Experimental Section

Chemicals. All chemicals were purchased unless otherwise indicated and were used as received without further treatment. Chemicals including molybdenum hexacarbonyl ($Mo(CO)_6$, ≥99.9%), palladium acetylacetonate ($Pd(acac)_2$, 99%), nickel(II) acetylacetonate ($Ni(acac)_2$, 95%), sulfur (99.998%), oleylamine (OAm, technical grade, 70%), trioctylphosphine (TOP, 97%), molybdenum(IV) disulfide powder (c-MoS_2, 98%), and ethanol (absolute, ≥99.8%) were purchased from Sigma-Aldrich. *n*-Hexane (≥97.0%) was ordered from Honeywell.

Synthesis of MoS_2/C and MoS_2 Catalysts. The synthesis of MoS_2 monolayers were conducted in an inert atmosphere using standard Schlenk techniques and the work-up procedures were carried out in air. Typically, a 250 mL round bottom three-neck flask containing 20 mL of OAm, 2.0 mmol of $Mo(CO)_6$ and 4.0 mmol of sulfur was heated up to 80 °C using a heating mantle (Glas-Col, LLC). The flask was connected to an argon gas inlet and a reflux condenser attached with a bubbler. After being deaerated with argon for at least 30 min, the reactants were rapidly heated up to 300 °C in 3 min. The reaction was held for 1 h and the flask was then cooled down naturally. The reaction medium was constantly stirred with a magnetic stir bar at 1000 rpm. Afterwards, a mixture of hexane and ethanol (v/v=1/2) was added to precipitate the products, and centrifugation was conducted to separate them at 15000 rpm for 5 min. The sediment was re-dispersed in 100 mL of hexane and 320 mg of Super P carbon black was added. The black suspension was sonicated for 2 h to load MoS_2 onto carbon support and the solvent was then slowly evaporated under vacuum at 40 °C. The product was again washed with a mixture of hexane and ethanol (v/v=1/5), and separated by centrifugation at 15000 rpm for 5 min. This washing process was repeated for 4 times in total. The final powder was collected by drying the sample in a vacuum oven at 40 °C overnight. Unsupported MoS_2 monolayers were directly recovered by washing for 4 times without adding carbon support.

The as-prepared MoS_2/C (designated as MoS_2/C-AP) or unsupported MoS_2 (designated as MoS_2-AP) was then calcined in a horizontal tube furnace at temperatures of 200, 300, 400, 500, and 600 °C (designated as MoS_2/C-*x* or MoS_2-*x*, *x* denotes the temperature). The powder was placed at the middle of a quartz tube, through which argon was flowing at a rate of 5 mL min^{-1}. The ramp rate was 5 °C min^{-1}. Each sample was kept at the targeted temperature for 4 h and then cooled down naturally.

Synthesis of Pd Nanoparticles. Pd nanoparticles were prepared using the modified organothermal decomposition method. All the reactions were conducted under inert atmosphere using standard Schlenk techniques and work-up procedures were carried out in air. Typically, a 250 mL round bottom three-neck flask containing 10 mL of OAm, 3 mL of TOP and 1.0 mmol of $Pd(acac)_2$ was heated up to 80 °C using a heating mantle (Glas-Col, LLC). The flask was connected to an argon gas inlet and a reflux condenser attached with a bubbler. After deaerating with argon for at least 30 min, temperature of the heating mantle was rapidly elevated to 230 °C in 3 min. The reaction was held for 1 h and the flask was then cooled down naturally. The reaction medium was constantly stirred with a magnetic stir bar at 1000 rpm. Afterwards, a

mixture of hexane and ethanol (v/v=1/2) was added to precipitate the products, and centrifugation was conducted to separate them at 15000 rpm for 5 min. The product was again washed with a mixture of hexane and ethanol (v/v=1/5), and separated by centrifugation at 15000 rpm for 5 min. This washing process was repeated for 4 times in total. The final powder was collected by drying the sample in a vacuum oven at 40 °C overnight.

Synthesis of Ni_2P Nanoparticles. Similarly, a 250 mL round bottom three-neck flask containing 10 mL of OAm, 10 mL of TOP and 1.0 mmol of $Ni(acac)_2$ was heated up to 80 °C using a heating mantle (Glas-Col, LLC). The flask was connected with an argon gas inlet and a reflux condenser attached with a bubbler. After deaerating with argon for at least 30 min, temperature of the heating mantle was rapidly elevated to 300 °C in 3 min. The reaction was held for 2 h and the flask was then cooled down naturally. The reaction medium was constantly stirred with a magnetic stir bar at 1000 rpm. Afterwards, a mixture of hexane and ethanol (v/v=1/2) was added to precipitate the products, and centrifugation was conducted to separate them at 15000 rpm for 5 min. The product was again washed with a mixture of hexane and ethanol (v/v=1/5), and separated by centrifugation at 15000 rpm for 5 min. This washing process was repeated for 4 times in total. The final powder was collected by drying the sample in a vacuum oven at 40 °C overnight.

Physical Characterization. The microstructures and morphology of the catalysts under study were characterized by transmission electron microscopy (TEM, JEOL JEM-2100F, 200 kV) and scanning electron microscopy (SEM, FEI Helios NanoLab 460HP, 10 kV). For the specimen preparation, one droplet of the sulfide suspension was dropped onto a copper grid coated with carbon film (400 mesh, TAAB) and dried in air.

X-ray powder diffraction (XRD) was performed to obtain the crystallographic information of the samples. The XRD patterns were recorded on a Malvern Panalytical X-ray diffractometer (Empyrean) using Cu Kα radiation source (λ = 1.5406 Å, 45 kV and 40 mA). Scanning angles for all samples were set in the 2θ range of 10−90° with a step size of 0.01313° and time per step of 120 s. Peaks were indexed according to the database established by Joint Committee on Powder Diffraction Standards (JCPDS).

Attenuated total reflection Fourier transform infrared (ATR-FTIR) spectroscopy was carried out on a Bruker VERTEX 70 spectrometer by using a DigiTect DLaTGS detector and platinum ATR. All spectra were recorded with a resolution of 2 cm^{-1} for 200 scans in the spectral range between $\tilde{\nu}$=400 and 4000 cm^{-1}. The background spectrum of air was measured as a single beam and used as reference.

Raman spectra were acquired using a confocal Raman microscope (SENTERRA II, Bruker) equipped with an optical microscope, a CCD camera, and an argon ion laser source. The laser provided 0.2 mW power at a wavelength of 532 nm for the exciting line. Integration time was 60 s, number of co-addition was 2, and slit aperture size was 50×1000 μm.

X-ray photoelectron spectroscopy (XPS) analysis was performed on the Nexsa X-ray photoelectron spectrometer system (Thermo Fisher Scientific) utilizing a monochromatic Al Kα source (1486.69 eV). High-resolution spectra were obtained at a pass energy of 40.0 eV, a step size of 0.1 eV, and a dwell time

of 50 ms per step. The analysis spot size was 100×200 μm. Depth-profiling XPS spectra were acquired by etching with 300 eV Ar^+ at a time interval of 45 s. All binding energies were calibrated to the graphitic C 1s peak at 284.5 eV or aliphatic C 1s peak at 285.0 eV. XPS peaks were deconvoluted and curve-fitted on the CasaXPS software using the Lorentzian function and Shirley background.

Rutherford backscattering spectrometry (RBS) was carried out at Dalton Cumbrian Facility, University of Manchester using a 5 MV tandem Pelletron ion accelerator and a TORVIS source. RBS spectra of the samples were acquired using a 4255 keV helium ion beam that was produced by plasma ionization, pre-acceleration, charge exchange, Pelletron acceleration, and argon stripping. The samples were dispersed on silicon wafers and mounted on a sample plate. The scattering angle of the RBS detector was 170°. The beam incident angle and exit angle were respectively 0 and 10° with a beam spot of 1.5 mm by 1.5 mm. The collected charge was set to 10 μC. Spectra simulation was performed using the SIMNRA code and the SigmaCalc scattering cross-sections. RBS spectra of graphite and gold film were measured for calibration.

Electrochemical Measurements. Electrochemical measurements were conducted on an Autolab PGSTAT101 potentiostat (Metrohm U.K. Ltd.) in a standard three-electrode cell at room temperature. A glass carbon electrode (GCE, diameter of 3 mm), an Ag/AgCl in 3 M KCl electrode and a platinum foil were used as working, reference and counter electrodes, respectively. 0.5 M H_2SO_4 was used as the electrolyte. All the potentials were calibrated to the reversible hydrogen electrode (RHE), according to Nernst equation $E_{vs\ RHE}=E_{vs\ Ag/AgCl}+0.21+0.0592\times pH$. Electrochemical impedance spectroscopy (EIS) was performed on a Reference 600+ (Gamry Instruments) potentiostat at a fixed overpotential of 300 mV vs RHE in the frequency range of 10^6 to 0.1 Hz. The measured potentials were corrected by iR_s compensation, where R_s was the solution resistance determined by the EIS. The geometric area of GCE (0.0707 cm^2) was used to calculate the current density in the linear sweep voltammetry (LSV).

Modified working electrode was fabricated via the following procedure: 5 mg of the as-prepared catalyst was dispersed in a mixture of 600 μL of DI water, 300 μL of ethanol and 100 μL of 5 wt % Nafion solution. The mixture was ultrasonically stirred for at least 2 h to obtain a homogeneous suspension. Then 3 μL of the suspension was taken by a microsyringe and drop-casted onto a clean GCE with an overall loading of 0.212 mg cm^{-2}. The modified GCE was dried naturally in air. Prior to the LSV test, the electrochemical cell was saturated by argon for at least 30 min and the modified GCE was activated by applying a cyclic voltammetry (CV) method in the potential range of 0 to 500 mV vs. RHE for 30 cycles at a scan rate of 200 mV s^{-1}. The LSV scan potential was set in the range of −600 to 100 mV vs. RHE and the scan rate was 5 mV s^{-1}.

Double-Layer Capacitance Calculation. To measure the double-layer capacitance, the potential range in which no apparent Faradaic processes occur is first determined from full cyclic voltammetry. This range is 0.25−0.35 V_{RHE}. All measured current in this non-Faradaic potential region is assumed to originate from

the double-layer capacitive behavior. The current density (j) equals to the product of the scan rate (v) and the electrochemical double-layer capacitance (C_{dl}), as given by the equation below.

$$j = v \times C_{dl}$$

The difference ($\Delta j_{0.3\ V}$) between the anodic charging and cathodic discharging current density measured at 0.3 V_{RHE} is used as j. Thus, a plot of $\Delta j_{0.3\ V}$ as a function of v yields a straight line with a slope equals to $2 \times C_{dl}$. The scan rates are in the range of 20−180 $mV \cdot s^{-1}$.

Note S1. Tafel analysis of HER kinetics

HER in acidic solution consists of three elementary steps: the Volmer, Heyrovsky and Tafel steps.

$$\text{(Volmer step)} \quad H_3O^+ + e^- + * \rightleftharpoons H^* + H_2O \tag{1}$$

$$\text{(Heyrovsky step)} \quad H^* + H_3O^+ + e^- \rightleftharpoons H_2 + H_2O + * \tag{2}$$

$$\text{(Tafel step)} \quad 2H^* \rightleftharpoons H_2 + 2* \tag{3}$$

where $*$ denotes the active site and H^* represent H atom adsorbed at the active site. The overall reaction rate can be determined by any of the three steps, namely the rate-determining step (RDS).

Here we define $r_i^{+/-}$ and $k_i^{+/-}$ as the reaction rate and reaction rate constant for the *i*th Equation. The $+$ and $-$ signs represent the forward and backward reactions, respectively. $[H_3O^+]$ defines the hydronium concentration. θ_* and θ_H define the surface coverage of free active site and hydrogen atom, respectively.

(1) Volmer step is the RDS

The forward and backward reaction rates of Volmer step can be expressed by Equations **4** and **5**.

$$r_1^+ = k_1^+[H_3O^+]\theta_* = k_1^+[H_3O^+](1-\theta_\mathrm{H}) \tag{4}$$

$$r_1^- = k_1^-\theta_\mathrm{H} = k_1^-(1-\theta_*) \tag{5}$$

When the Volmer step is the RDS, the HER reaction rate is determined by the forward reaction in Equation **1**. Since the Volmer step involves electron transfer, the rate constant is thus correlated to the applied potential, as in Equations **6** and **7**.

$$k_1^+ = k_1^{+0}e^{-\frac{\alpha_1 F}{RT}\eta} \tag{6}$$

$$k_1^- = k_1^{-0}e^{\frac{(1-\alpha_1)F}{RT}\eta} \tag{7}$$

where k_i^0 is the standard rate constant and α_i is the electron transfer coefficient for the *i*th Equation, η is the overpotential (difference between applied and standard potentials), F is Faraday constant (96,485 C·mol^{-1}), R is ideal gas constant (8.314 J·K^{-1}·mol^{-1}), and T is temperature in kelvin. Assuming the Volmer step as the RDS leads to the relatively fast Heyrovsky and Tafel steps, meaning that the adsorbed hydrogen is rapidly reacted and the θ_H is close to zero. Therefore, according to Equations **4** and **5**, the HER reaction rate can be described by Equation **8** (neglecting the contribution of backward reaction rate).

$$r_1^+ = k_1^{+0}[H_3O^+]e^{-\frac{\alpha_1 F}{RT}\eta} \tag{8}$$

The correlation between measured electric current, current density and reaction rate is described by Equations **9** and **10**.

$$i = nFAr \tag{9}$$

$$j = nFr \tag{10}$$

where i is the electric current, j is the current density, n is the number of transferred electrons, and A is the surface area of catalyst. Combining Equations **8** and **9** gives the current density by Equation **11** (neglecting the contribution of backward reaction rate).

$$j_{RDS} = nFk_1^{+0}[H_3O^+]e^{-\frac{\alpha_1 F}{RT}\eta} \tag{11}$$

According to the Tafel equation, $\eta = a + blogj$, Tafel slope is thus derived from Equation **11**.

$$b = \frac{2.303RT}{\alpha_1 F} \tag{12}$$

α_1 equals to 0.5, then b is **~118 mV decade^{-1}**.

(2) Heyrovsky step is the RDS

When the Heyrovsky step is the RDS, the Volmer step shall be pre-equilibrated, meaning that the forward and backward reaction rates in Equations **4** and **5** are equal to each other.

$$r_1^+ = k_1^{+0}e^{-\frac{\alpha_1 F}{RT}\eta}[H_3O^+](1-\theta_{\mathrm{H}}) = r_1^- = k_1^{-0}e^{\frac{(1-\alpha_1)F}{RT}\eta}\theta_{\mathrm{H}} \tag{13}$$

The hydrogen coverage is thus described by Equation **14**.

$$\theta_{\mathrm{H}} = \frac{K_1^0[H_3O^+]}{K_1^0[H_3O^+]+e^{\frac{F}{RT}\eta}} = xe^{-\frac{F}{RT}\eta} \tag{14}$$

where standard equilibrium constant K_i^0 is defined by the ratio k_i^{+0}/k_i^{-0} and x is a coefficient to describe the dependence of θ_{H} on the monomial $e^{\frac{F}{RT}\eta}$.

The forward reaction rate of the Heyrovsky step (Equation **2**) is given by Equation **15**.

$$r_2^+ = k_2^+[H_3O^+]\theta_H = k_2^{+0}e^{-\frac{\alpha_2 F}{RT}\eta}[H_3O^+]\theta_H \tag{15}$$

Combining Equations **10**, **14** and **15** gives the electric current by Equation **16** (neglecting the contribution of backward reaction rate).

$$j_{RDS} = nFr_2^+ = nFk_2^{+0}[H_3O^+]xe^{-\frac{(1+\alpha_2)F}{RT}\eta} \tag{16}$$

Tafel slope is thus given by Equation **17**.

$$b = \frac{2.303RT}{(1+\alpha_2)F} \tag{17}$$

α_2 also equals to 0.5, then b is **~39 mV decade^{-1}**.

(3) Tafel step is the RDS

When the Tafel step is the RDS, the pre-equilibrium of Volmer step is also established and Equations **13** and **14** are still tenable. The forward reaction rate of the Tafel step (Equation **3**) is given by Equation **18**.

$$r_3^+ = k_3^+ {\theta_\mathrm{H}}^2 = k_3^{+0} {\theta_\mathrm{H}}^2 \tag{18}$$

Combining Equations **10**, **14** and **18** gives the electric current by Equation **19** (neglecting the contribution of backward reaction rate).

$$j_{RDS} = nFr_3^+ = nFk_3^{+0} x^2 e^{-\frac{2F}{RT}\eta} \tag{19}$$

Tafel slope is given by Equation **20**.

$$b = \frac{2.303RT}{2F} \tag{20}$$

Therefore b is **~30 mV decade^{-1}**.

Note S2. Rutherford backscattering spectrometry (RBS)

As part of the high energy ion beam analysis, RBS measures the energy loss of incident particles (projectiles) after the elastic collision with initially stationary target atoms (targets), as illustrated in the figure on the right. The masses and mass numbers of projectile A and target B are M_A, Z_A and M_B, Z_B. Initial velocity and energy of projectile A are $\mathbf{v}_{A0}$ and E_{A0}. After collision, the velocity and energy of projectile A and target B are $\mathbf{v}_{A1}$, E_{A1} and $\mathbf{v}_{B1}$, E_{B1}.

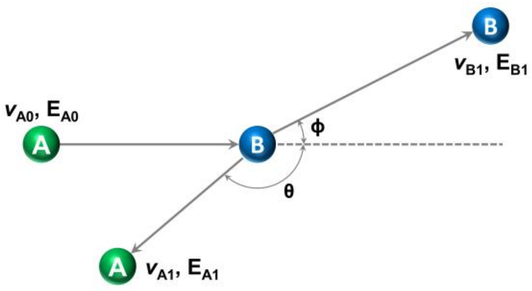

(1) Kinematic factor K

Conservation of energy and momentum are expressed by the Equations **22** and **23** below:

$$\frac{1}{2}M_A {v_{A0}}^2 = \frac{1}{2}M_A {v_{A1}}^2 + \frac{1}{2}M_B {v_{B1}}^2 \tag{22}$$

$$M_A \overrightarrow{v_{A0}} = M_A \overrightarrow{v_{A1}} + M_B \overrightarrow{v_{B1}} \tag{23}$$

The ratio of the projectile energy after collision to that before the collision is defined as the *kinematic factor K*.

$$K = \frac{E_{A1}}{E_{A0}} \tag{24}$$

From Equations **22** and **23** we obtain Equation **25**.

$$K = \left[\frac{M_A \cos\theta \pm \sqrt{{M_B}^2 - {M_A}^2 sin^2\theta}}{M_A + M_B}\right]^2 \tag{25}$$

For $M_A \leq M_B$, the plus sign applies, which is also the case of RBS as light ion (e.g., proton and helium ions) beams are often used, otherwise the minus sign is taken. Therefore, one can identify the target atoms by measuring the energy of backscattered projectiles at a given scattering angle.

If the energy of incident projectile is high enough to overcome the Coulomb barrier, nuclear reactions may occur, despite that the collision remains elastic. In this case, it is termed as non-Rutherford elastic backscattering spectrometry (EBS).

(2) Scattering cross section σ

Albeit the establishment of collision energetics above, one has to refer to the differential scattering cross section σ to answer the question on how frequently the collision actually occurs and leads to the scattering at a specific angle. The differential scattering cross section σ is defined as

$$\frac{d\sigma}{d\Omega} = \frac{1}{\rho l Q}\frac{dQ}{d\Omega} \tag{26}$$

where ρ is the volume density of target atoms, l is the thickness, Q is the total number of projectiles that hit the target, dQ is the number of particles recorded by the detector in the differential solid angle $d\Omega$. ρl is thus the areal density.

For an elastic collision, the differential scattering cross section σ in the laboratory frame of reference is generally given by

$$\frac{d\sigma}{d\Omega} = \left(\frac{Z_A Z_B e^2}{4E_A}\right)^2 \frac{4}{sin^4\theta} \frac{\left\{\sqrt{1-\left(\frac{M_A}{M_B}\sin\theta\right)^2}+\cos\theta\right\}^2}{\sqrt{1-\left(\frac{M_A}{M_B}\sin\theta\right)^2}} \propto \left(\frac{Z_A Z_B e^2}{4E_A}\right)^2 \tag{27}$$

where E_A is the energy of the projectile immediately before collision. From Equation **27**, one can find that the backscattering yield is higher with heavier projectiles for the same target. Heavier targets have higher probability to scatter lighter projectiles. Yield of scatter projectiles increases with decreasing bombarding energy/slower projectiles. Furthermore, the scattering cross section σ deviates from that of RBS for the EBS.

(3) Energy loss by thickness effect

In the case of an energetic beam bombarding a target, the primary process is the implantation of the projectiles into the target, while the large-angle Rutherford scattering is indeed a secondary process. As the projectile travels through the target, its kinetic energy decreases. The amount of energy loss ΔE per traversed distance Δx depends on the nature of the projectile, on the density and composition of the target, and on the velocity itself. Compared to the topmost surface targets, targets located at a certain depth to the surface are impinged by lower energy projectiles and thus scattering yield is higher than that at the surface. Accordingly, depth profile of the target can be obtained.

(4) Thin film unit (TFU)

In backscattering analysis, thickness is given by energy loss from thin film samples whose thickness is determined by mass per unit area, or areal density. Units of eV/(10^{15} atoms·cm^{-2}) are used for the energy loss database where 10^{15} atoms·cm^{-2} is a thickness of the order of a monolayer since most materials have an atom density in the order of 10^{22} atoms·cm^{-3}. 1 TFU is defined as 10^{15} atoms·cm^{-2}. Given the atom density, TFU is then equivalent to linear units of thickness (nm). For amorphous carbon, MoS_2 and silicon with densities of 2.0, 5.1 and 2.3 g·cm^{-3}, 10 TFU roughly correspond to 1.0, 1.7 and 1.4 nm.

Table S1. Normalized elemental contents in atomic percentage of the calcined MoS_2 and MoS_2-AP derived from the RBS analysis.

**Samples**	**Elemental content (atom %)**		
	**C**	**Mo**	**S**
**MoS_2-AP**	77.7	8.1	14.2
**MoS_2-200**	75.9	8.2	15.9
**MoS_2-300**	69.3	10.6	20.1
**MoS_2-400**	55.4	14.8	29.8
**MoS_2-500**	56.9	15.7	27.4
**MoS_2-600**	59.8	14.5	25.7

Table S2. Normalized elemental contents in atomic percentage of the calcined MoS_2 and MoS_2-AP derived from the depth-profiling XPS after 90s etching.

Samples	Elemental content (atom %)		
	C	Mo	S
**MoS_2-AP**	93.97	3.07	2.96
**MoS_2-200**	91.49	3.26	5.25
**MoS_2-300**	78.47	8.32	13.21
**MoS_2-400**	69.98	10.40	19.62
**MoS_2-500**	72.17	10.96	16.87
**MoS_2-600**	76.86	8.83	14.31

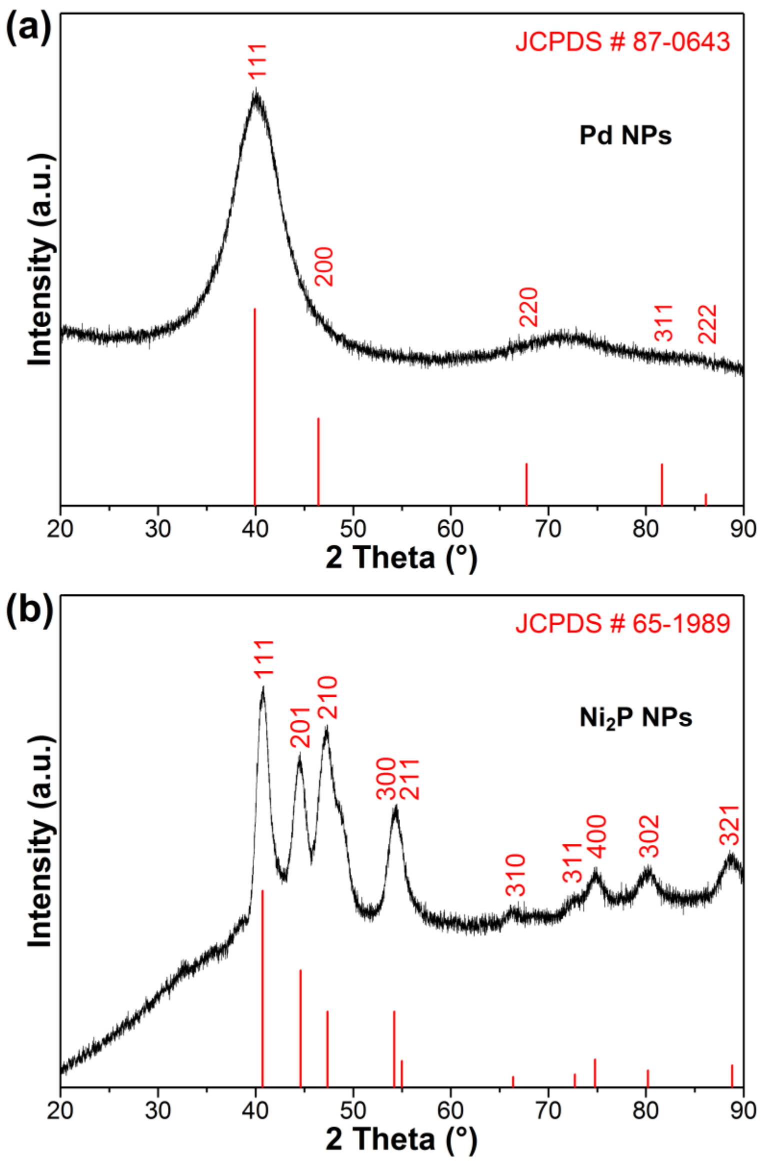

Figure S1. XRD patterns of the as-prepared Pd (a) and Ni_2P (b) NPs indexed to JCPDS card No. 87-0643 and 65-1989, respectively.

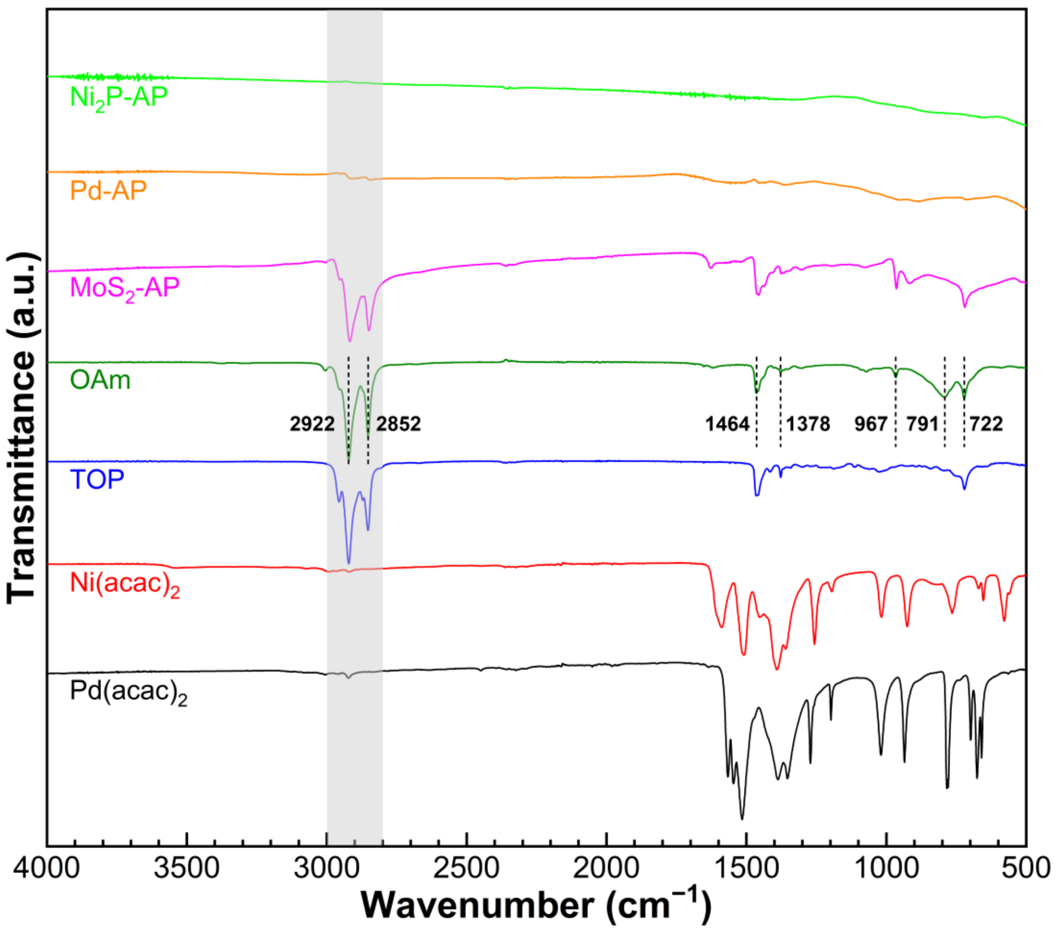

Figure S2. ATR-FTIR spectra of the as-prepared Pd and Ni_2P NPs, MoS_2-AP, and reference chemicals of $Pd(acac)_2$, $Ni(acac)_2$, TOP, and OAm.

The absorption peaks at the wavenumber of 2922, 2852, 1464, 1378, 967, 791, and 722 cm^{-1} correspond to the C–H asymmetric stretching, the C–H symmetric stretching, the C–H asymmetric bending, the C–H symmetric bending, the $-NH_2$ bending, the C=C stretching, and the methylene rocking. The most representative C–H stretching peaks at 2922 and 2852 cm^{-1} are highlighted.

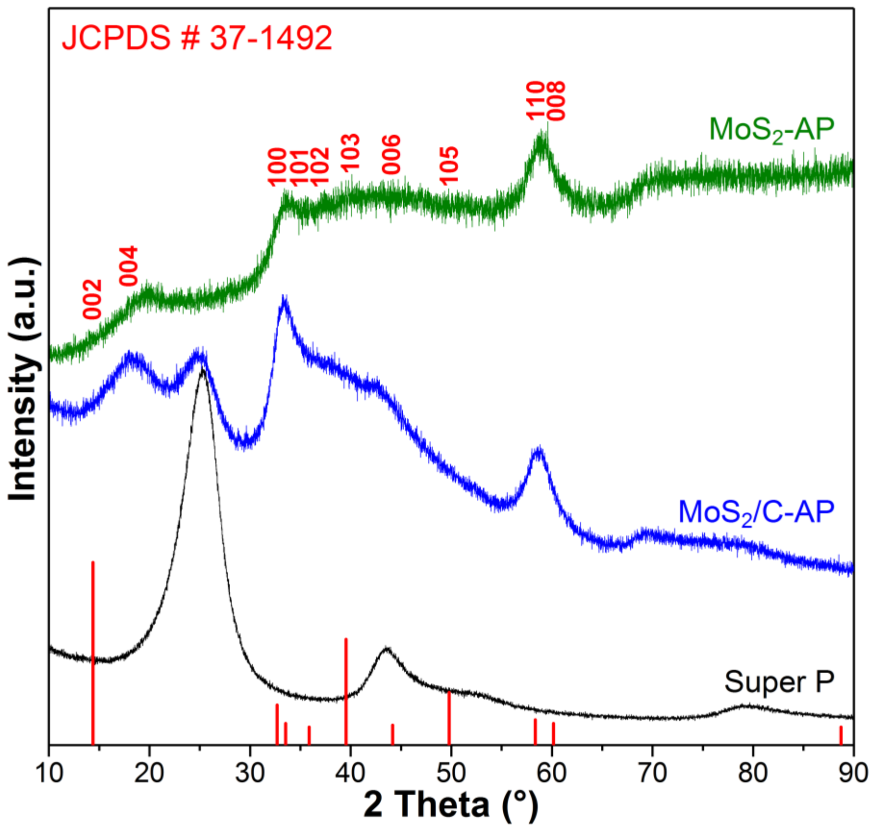

Figure S3. XRD patterns of Super P, MoS_2/C-AP, and MoS_2-AP indexed to the hexagonal 2H-MoS_2 with the JCPDS card No. 37-1492.

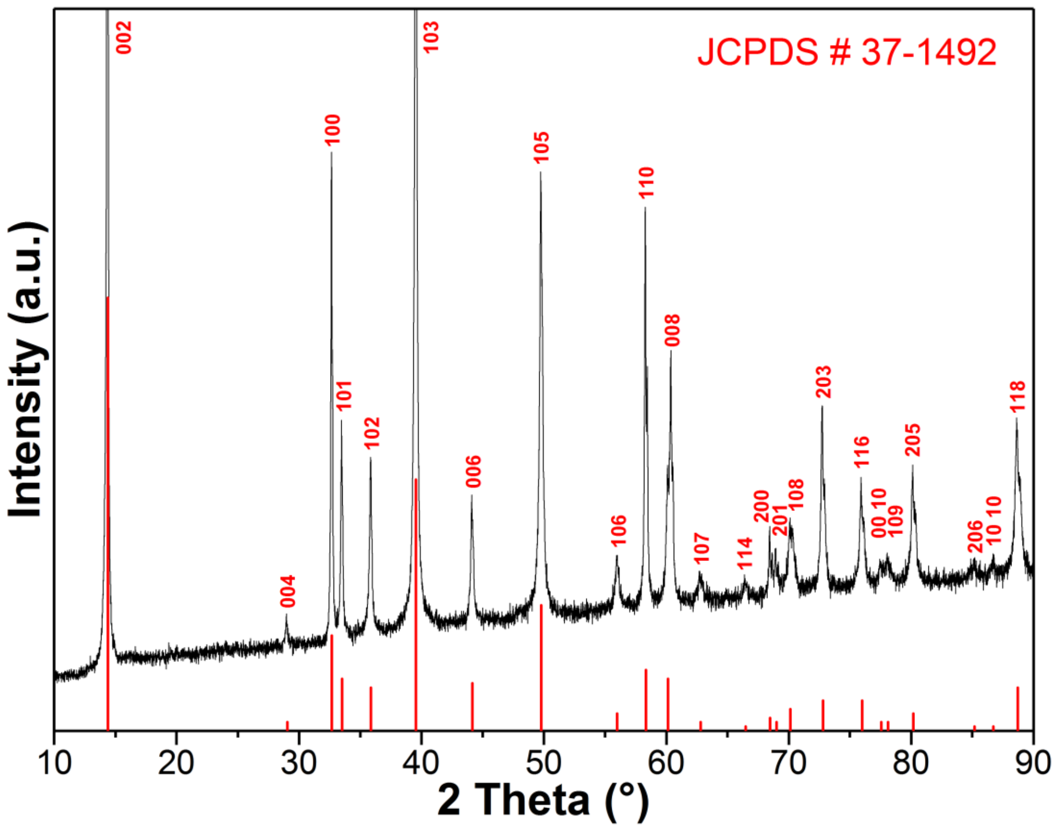

Figure S4. XRD pattern of the c-MoS_2 indexed to the hexagonal 2H-MoS_2 with the JCPDS card No. 37-1492.

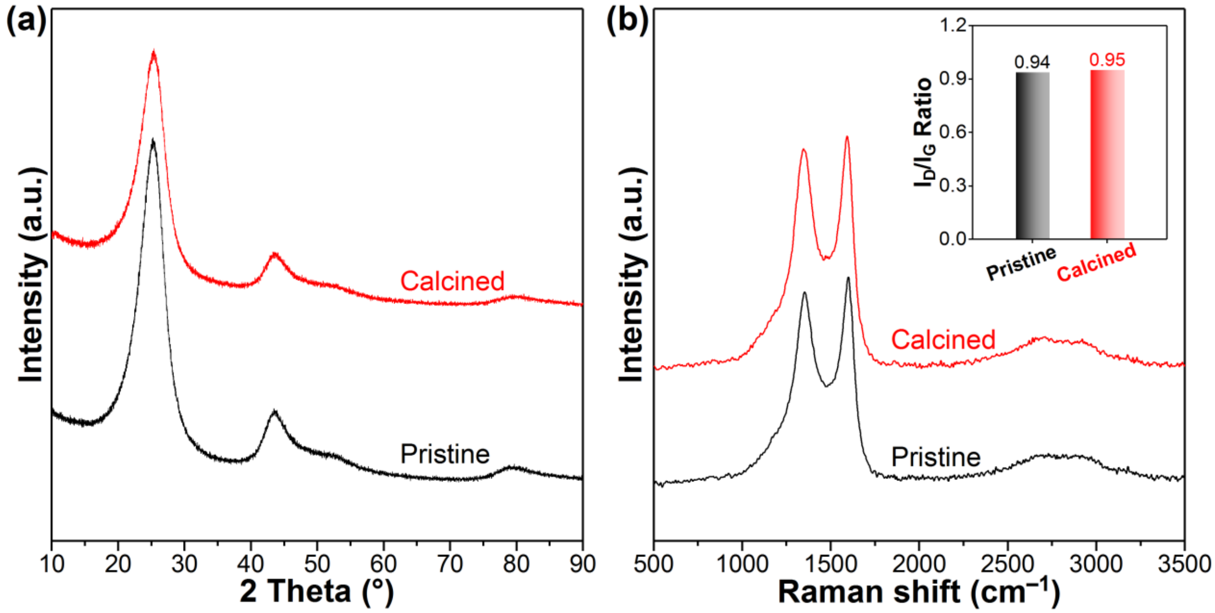

Figure S5. XRD patterns (a) and Raman spectra (b) of pristine and calcined Super P carbon black at 600 °C for 4 h in Ar.

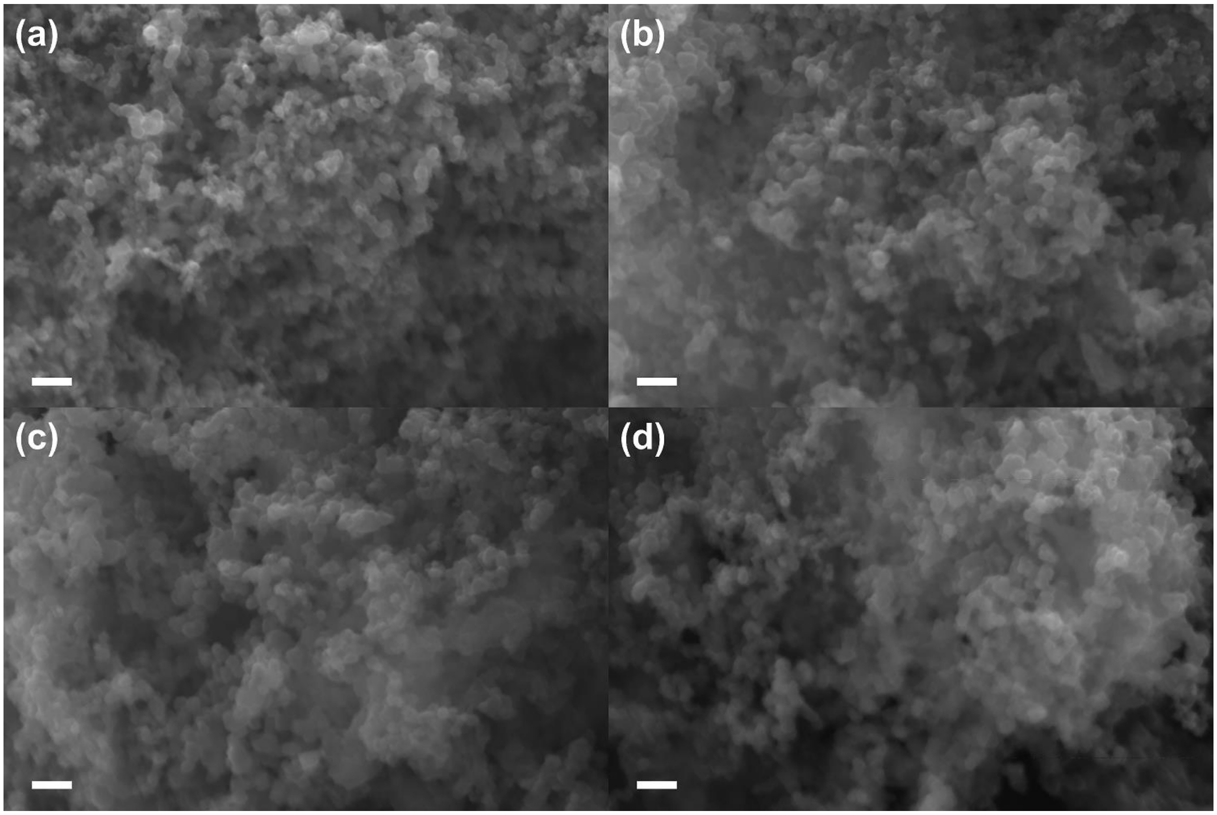

Figure S6. SEM images of Super P (a), MoS_2/C-AP (b), MoS_2/C-400 (c), and MoS_2/C-600 (d). Scale bar: 100 nm.

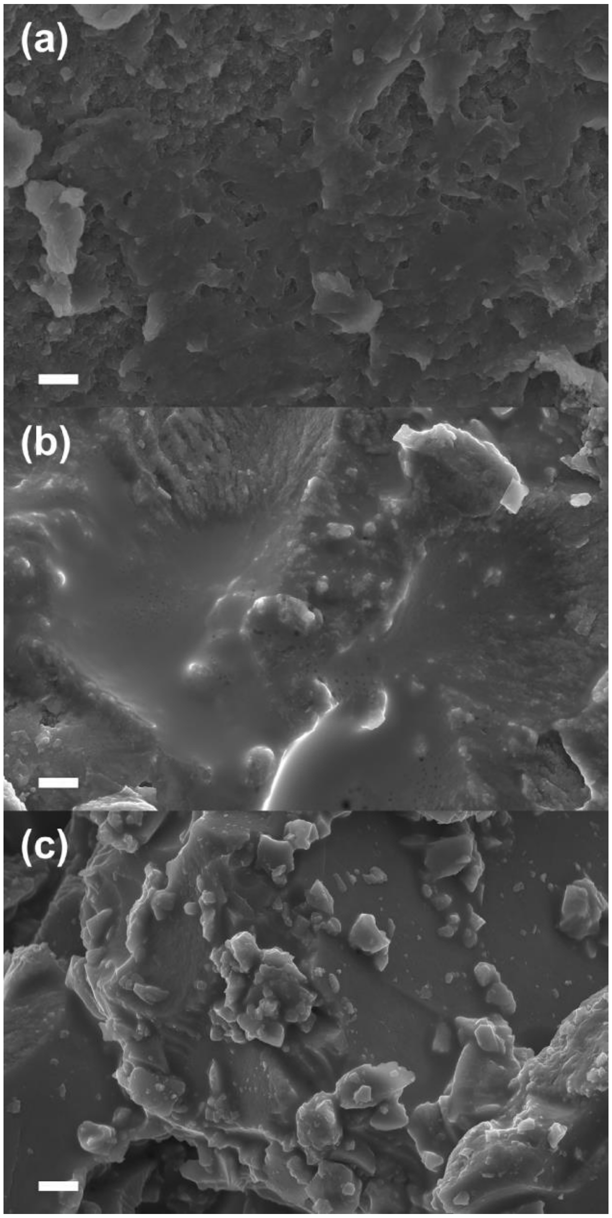

Figure S7. SEM images of MoS_2-AP (a), MoS_2-400 (b), and MoS_2-600 (c). Scale bar: 1 μm.

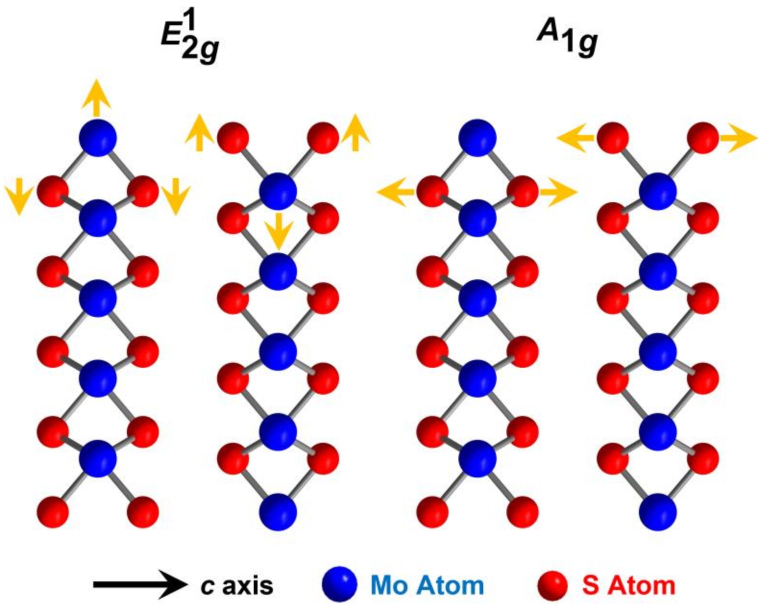

Figure S8. Schematic illustration of the $E_{2g}{}^1$ and A_{1g} vibrational modes of MoS_2.

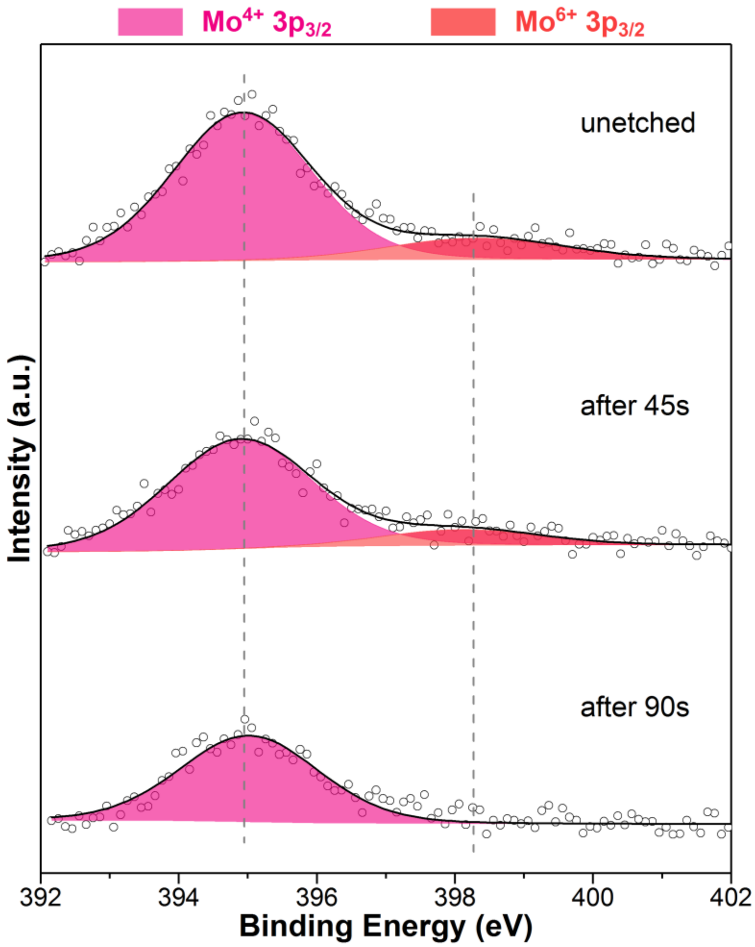

Figure S9. Depth-profiling XPS spectra of the Mo 3p region of c-MoS_2 etched by 300 eV Ar^+: unetched (top), after 45 s etching (middle), and after 90 s etching (bottom). The same intensity scale is set for all the samples.

Upon Ar^+ etching, the oxidized Mo is removed, as indicated by the disappearance of Mo^{6+} $3p_{3/2}$ species after 90 s.

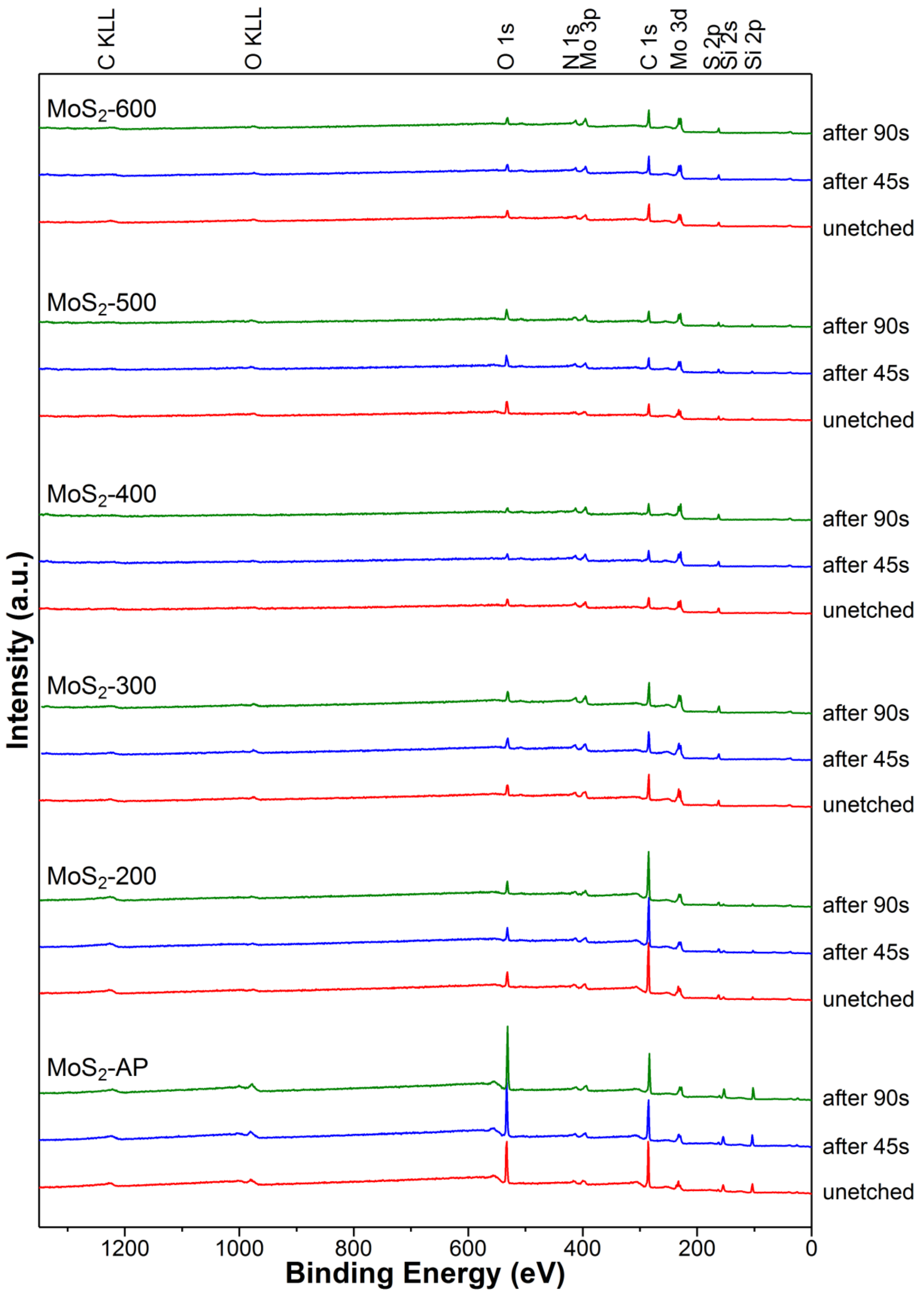

Figure S10. Depth profiling XPS full survey of the calcined MoS_2 etched by 300 eV Ar^+. The red, blue, and green lines represent unetched, after 45s etching, and after 90s etching, respectively.

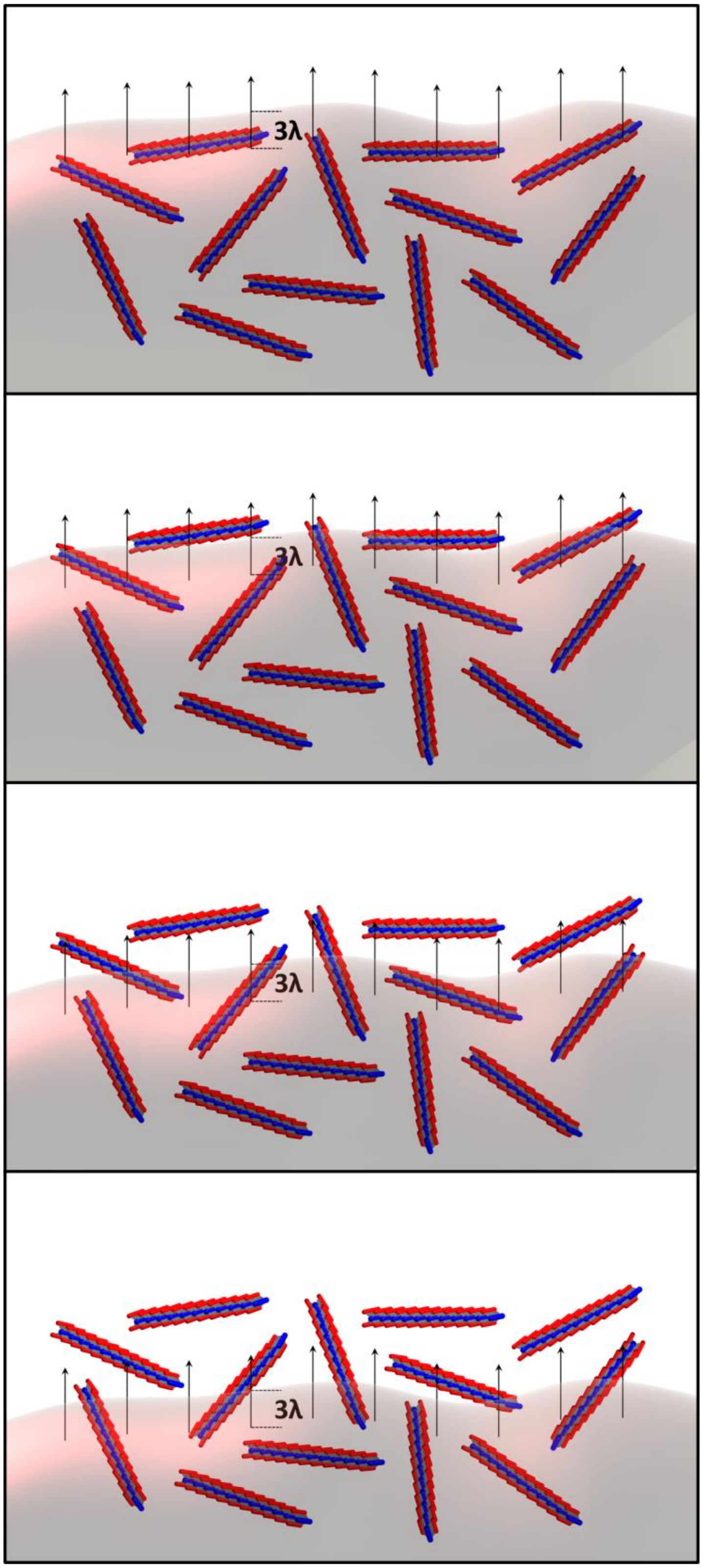

Figure S11. Schematic illustration of the encapsulated MoS_2 monolayers randomly oriented and distributed within the C shell. The depth of 3λ (λ is the electron mean free path) that most photoelectrons can escape and be detected by XPS is marked.

As the topmost surface layer is etched away gradually, new surface with statistically the same C content is then exposed, accounting to the inconspicuous C 1s peak changes.

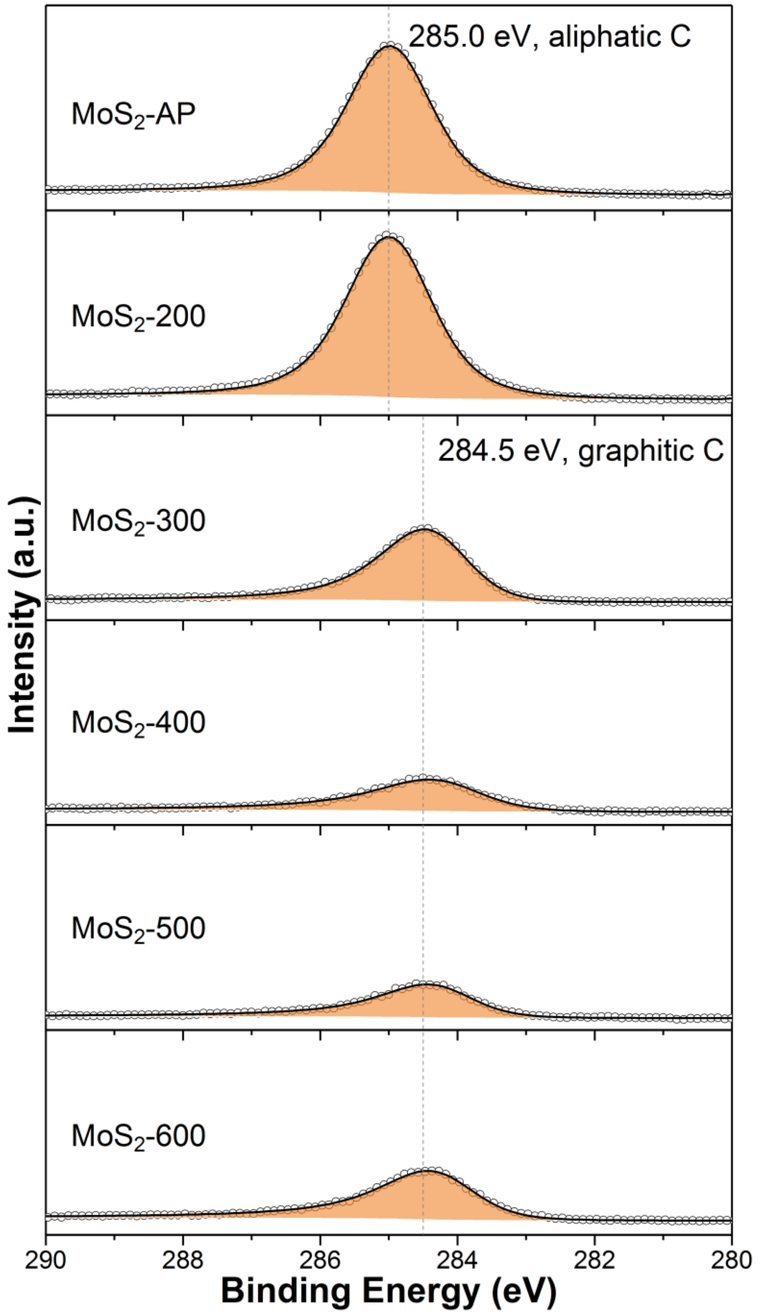

Figure S12. High-resolution XPS spectra of C 1s region of the calcined MoS_2 etched by 300 eV Ar^+ for 90 s for removal of adventitious carbon. The same intensity scale is set for all of the samples.

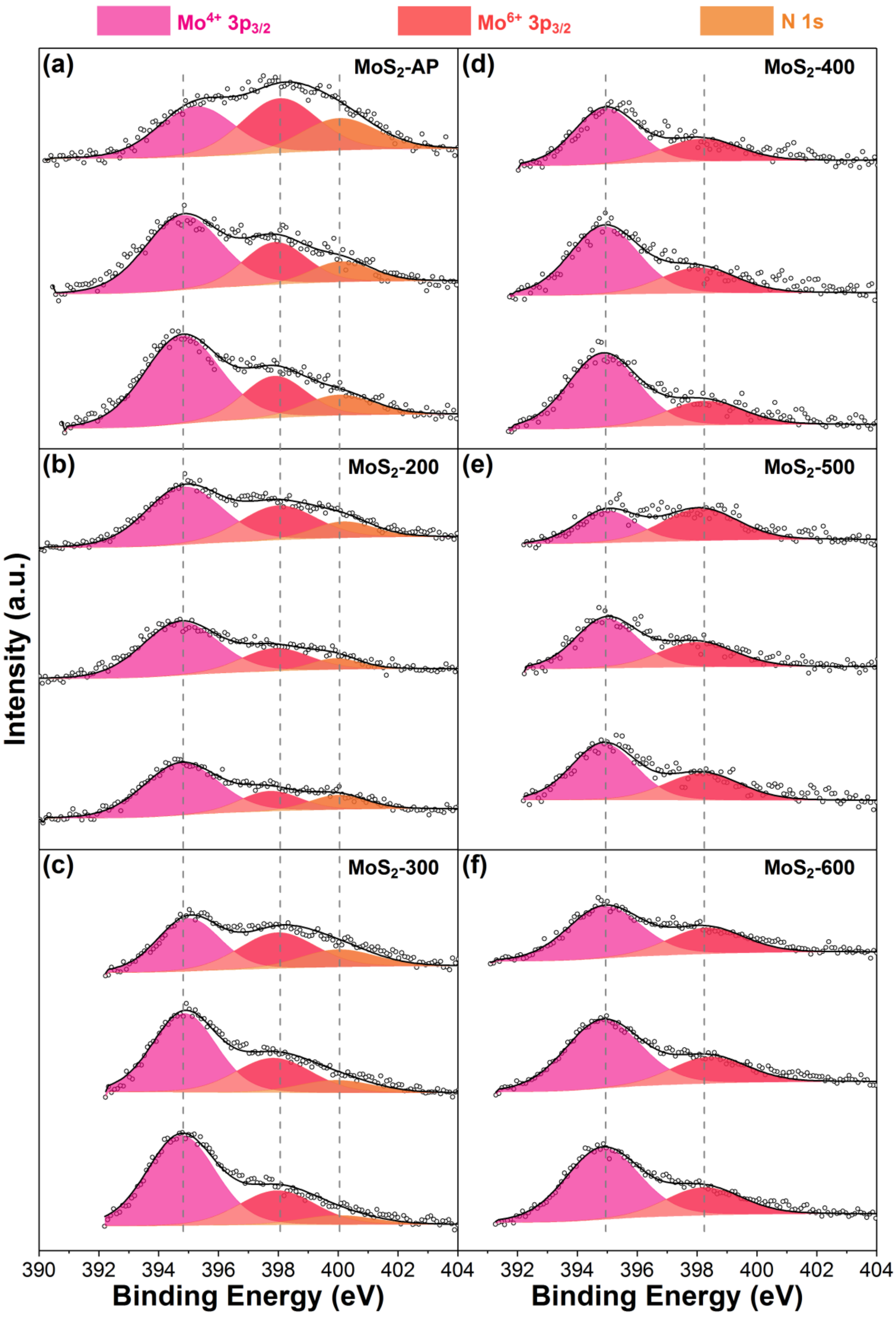

Figure S13. Depth profiling XPS spectra of the Mo 3p/N 1s region of the calcined MoS_2 etched by 300 eV Ar^+. In each panel, the top, middle, and bottom spectra represent unetched, after 45 s etching, and after 90 s etching, respectively.

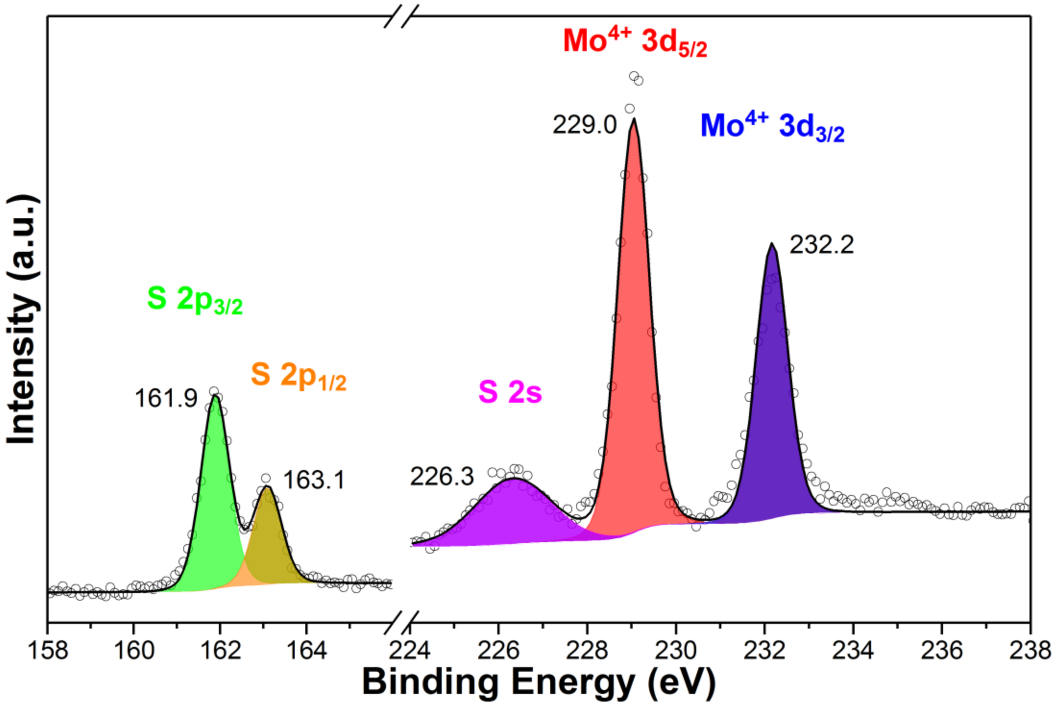

Figure S14. High-resolution XPS spectra of Mo 3d and S 2p regions of c-MoS_2 after 90s etching by Ar^+. Mo 3d peak is resolved into spin-orbit doublets, Mo^{4+} $3d_{5/2}$ and $3d_{3/2}$, at 229.0 and 232.2 eV. S 2p peak is resolved into spin-orbit doublets, S $2p_{3/2}$ and $2p_{1/2}$, at 161.9 and 163.1 eV.

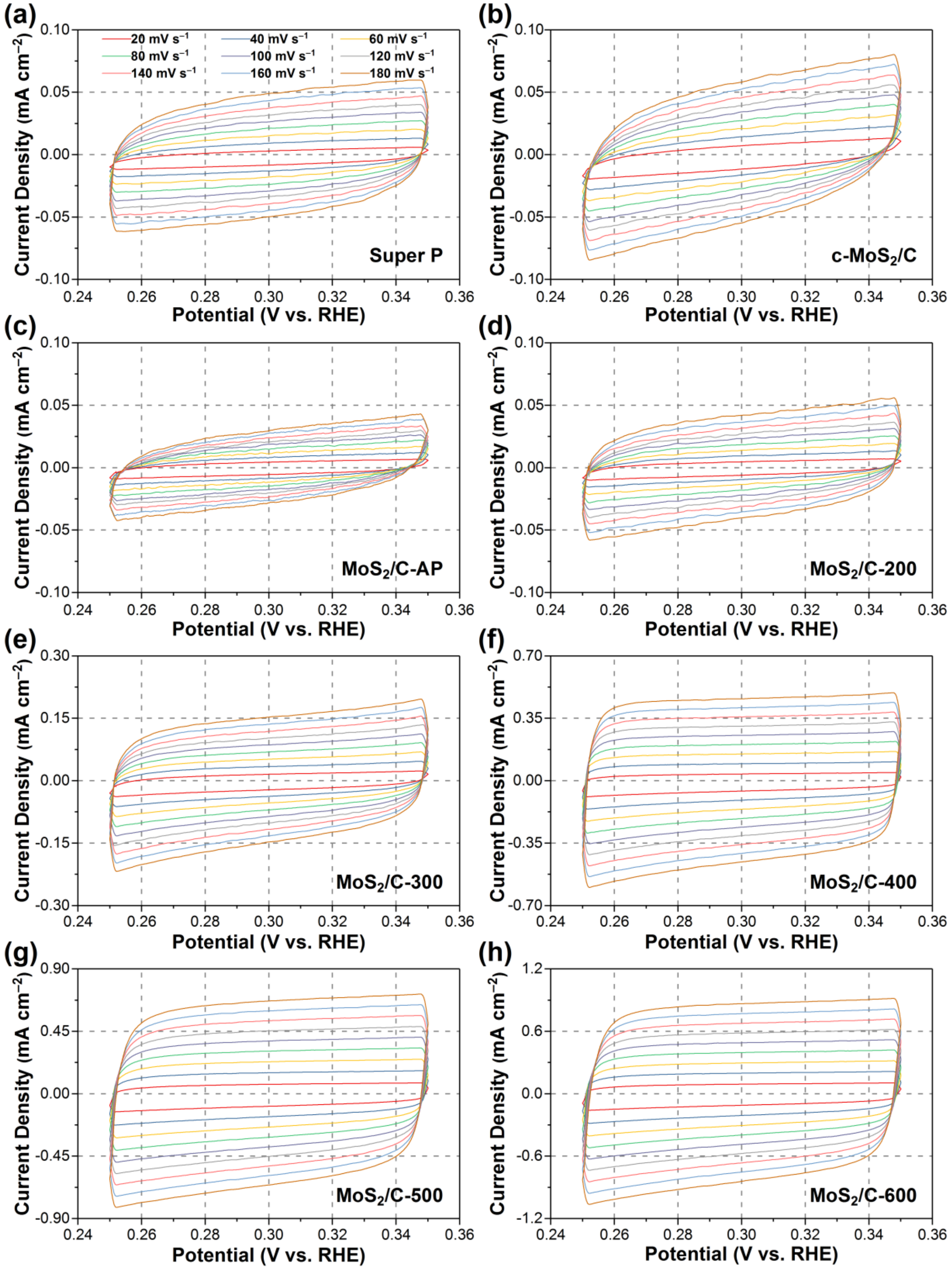

Figure S15. Cyclic voltammograms of Super P (a), c-MoS_2/C (b), MoS_2/C-AP (c) and MoS_2/C calcined at temperatures of 200 (d), 300 (e), 400 (f), 500 (g), and 600 (h) °C in the potential range of 0.25–0.35 V_{RHE} and the scan rate range of 20–180 mV s^{-1}.

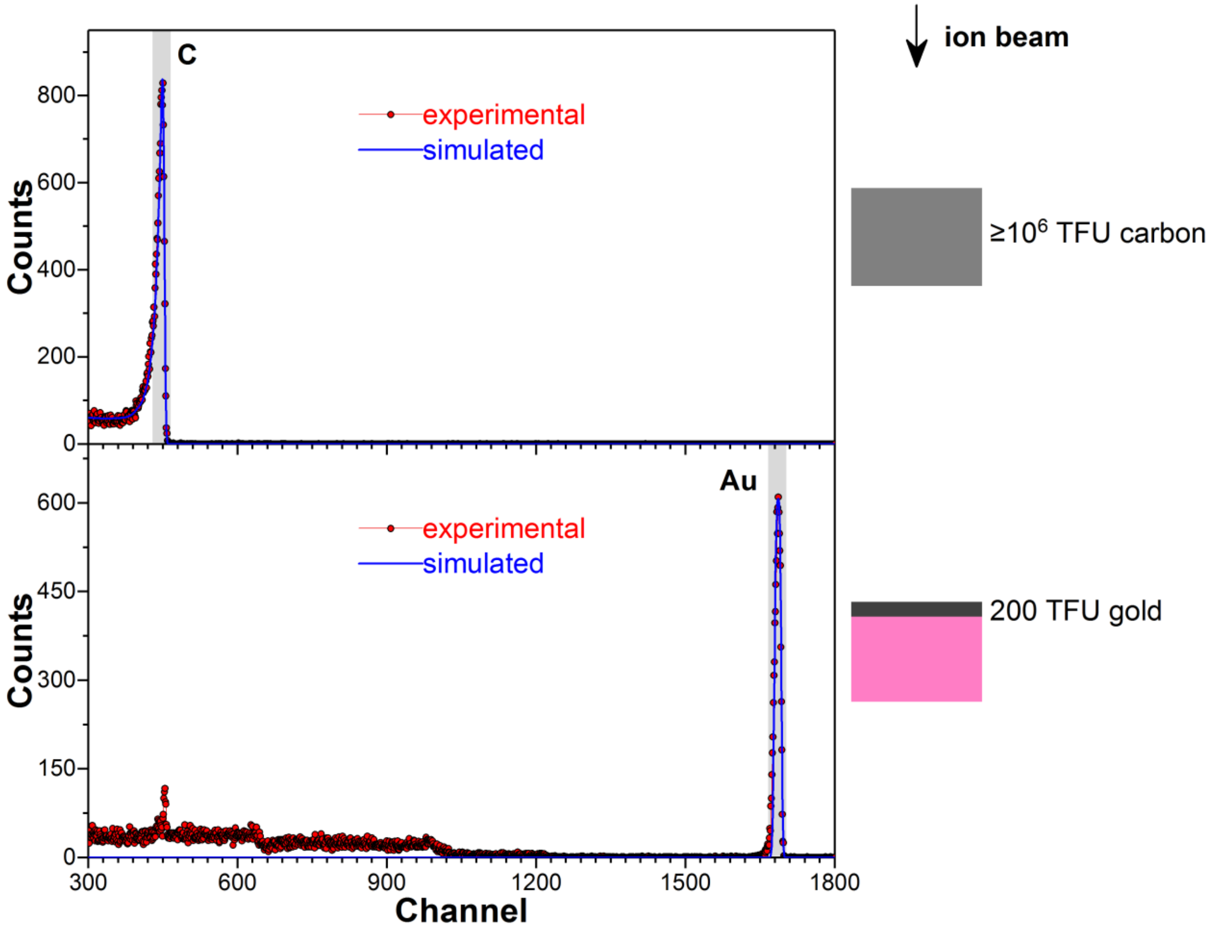

Figure S16. Experimental and simulated RBS spectra of thick graphite and thin gold film references. The areal thickness is indicated on the right. The substrate of gold film is not simulated.

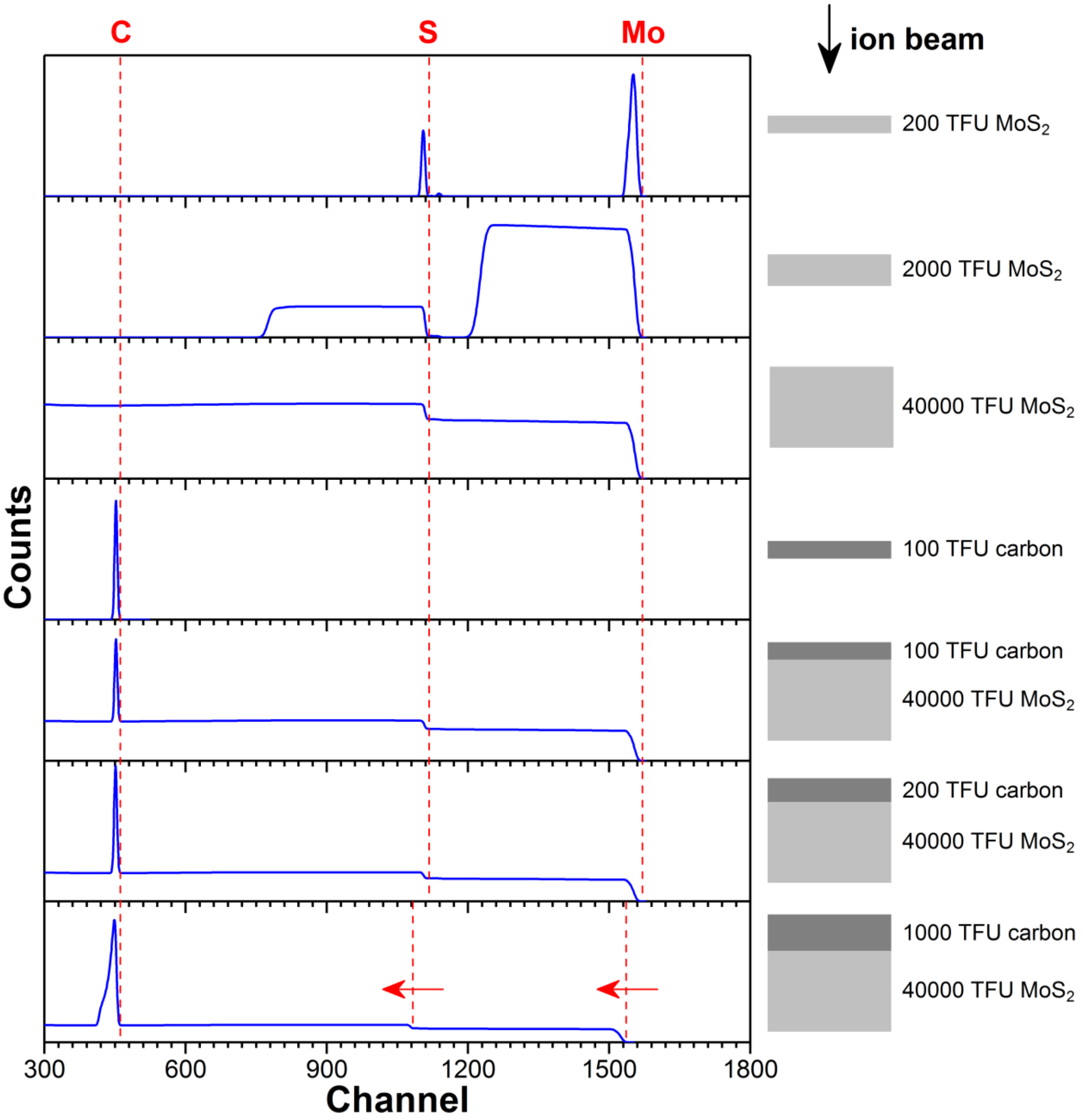

Figure S17. Simulated RBS spectra of MoS_2 films with varied thicknesses of 200, 2000, and 40000 TFU, carbon film with a thickness of 100 TFU, and carbon films with thicknesses of 100, 1000 TFU on top of a 40000 TFU MoS_2 film.

For a thin film of MoS_2 or carbon, individual sharp peaks of Mo, S and C elements are observed. The peaks widen with the formation of plateau on the top as the thickness increases. The front edge of each element corresponding to scattering by the topmost surface atoms should be identical. The back edge of the peak is due to scattering by atoms located at the backside. When a thin carbon layer is deposited on MoS_2, projectiles have to travel through the carbon film before bombarding the MoS_2 atoms and the kinetic energy of projectiles is thus decreased. This results in the shift of the front edges of Mo and S to lower energies compared to the uncovered MoS_2.

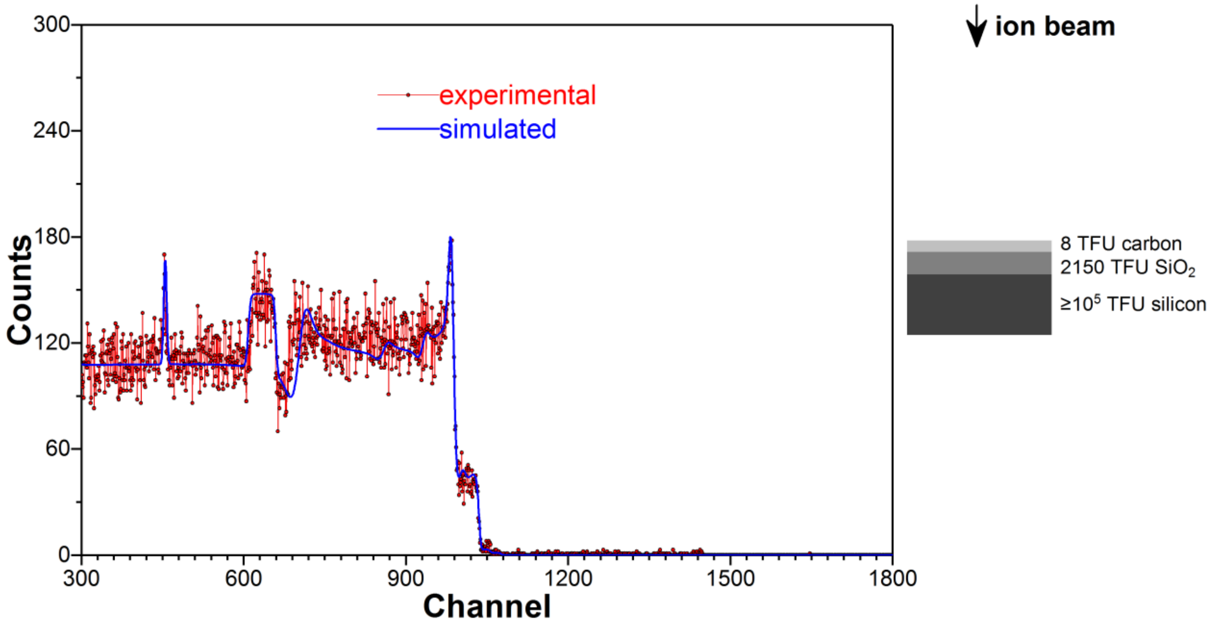

Figure S18. Experimental and simulated RBS spectra of blank silicon wafer deposited by pure solvent. The areal thickness is indicated on the right. Only tiny amount of adventitious carbon is detected.
